# Maternal probiotic mixture supplementation optimizes the gut microbiota structure of offspring piglets through the gut–breast axis

**DOI:** 10.1016/j.aninu.2024.04.025

**Published:** 2024-07-17

**Authors:** Ting Hu, Zhiguan Song, Lan Yang, Keyuan Chen, Yi Wu, Fei Xie, Jiufeng Wang, Guiyan Yang, Yaohong Zhu

**Affiliations:** a College of Veterinary Medicine, China Agricultural University, Beijing 100193, China; b Sanya Institute of China Agricultural University, Sanya 572025, China; c Animal Disease Prevention and Control Center of Shanxi Province, Taiyuan 030027, China; d Beijing Da Bei Nong Science and Technology Group Co., Ltd., Beijing 100192, China

**Keywords:** Probiotic mixture, Gut microbiota, Colostrum metabolome, Gut–breast axis, Sow, Piglet

## Abstract

Delivery and weaning are major stressful events in sows and piglets, adversely affecting production and growth performance and causing economic losses to swine farms. Probiotics as safe antibiotic alternatives have great potential for use across all stages of livestock farming. Here, 18 pregnant sows from clinical farms randomly were divided into two groups: one fed a basal diet (CON group) and the other fed a basal diet plus a probiotic mixture CBB-mix (containing 1×10^12^ CFU/g of *Lactobacillus johnsonii* [CJ21], 1×10^9^ CFU/g of *Bacillus subtilis* [BS15], and 1×10^9^ CFU/g of *Bacillus licheniformis* [BL21]), for 20 days before delivery. The effects of maternal CBB-mix supplementation on sow colostrum metabolome and offspring piglets' clinical performance, immune status, and gut microbiota were investigated. Additionally, 177 piglets were randomly divided into 4 groups, including CC group (piglets and sows fed a basal diet, *n* = 40 from 5 litters), CP group (piglets fed the basal diet plus CBB-mix and sows fed the basal diet, *n* = 38 from 4 litters), PC group (piglets fed the basal diet and sows fed the basal diet plus CBB-mix, *n* = 50 from 4 litters), and PP group (both piglets and sows fed the basal diet plus CBB-mix, *n* = 49 from 5 litters). Among that, CP and PP groups were added CBB-mix in the creep feed from 11 days of age for 18 days to study the direct effects of CBB-mix on the growth performance of piglets. Maternal CBB-mix supplementation improved sow production performance, including litter size at birth and litter weight at birth (*P* < 0.05). Piglets born from CBB-mix-fed sows exhibited increased litter size at weaning and reduced diarrhea incidence from 1 to 10 days of age (*P* < 0.05). Additionally, systemic immune status and antioxidant capabilities were improved in both sows and piglets. Maternal CBB-mix supplementation reconstituted the gut microbiota structure and increased the Sobs index and Shannon index of fecal microbiota in both sows and piglets (*P* < 0.05). The relative abundance of Firmicutes and *Clostridium_sensu_stricro_1* in sow feces was decreased after feeding CBB-mix (*P* < 0.05). In piglets, 10-day-old feces had relatively more *Lactobacillus* but less *Escherichia-Shigella* than 1-day-old feces (*P* < 0.05), indicating that maternal feeding CBB-mix alone affects the gut microbiota community of offspring piglets via the gut–breast axis. Piglets born from CBB-mix-fed sows had continuously decreased the relative abundance of fecal *Escherichia-Shigella* at 28 days of age (*P* < 0.05). Consistently, the metabolite profile in sow milk was also changed by CBB-mix. Colostrum metabolome showed that CBB-mix significantly regulated tryptophan metabolism and primary bile acid biosynthesis. Our data demonstrated that maternal CBB-mix supplementation effectively improved the production performance of sows and their offsprings' growth performance. Through the gut–breast axis (interaction between gut microbiota and mammary glands), feeding CBB-mix to sows impacted the gut microbiota of their offspring. This study provides strategy and evidence for maternal probiotic supplementation to improve immune status and gut microbiota homeostasis in response to delivery and weaning.

## 1 Introduction

Parturition and weaning are major stressful events in the lives of sows and piglets, respectively. The postpartum period for sows may entail various health challenges, including constipation, postpartum inflammation, decreased appetite, and insufficient lactation due to farrowing stimulation (Quesnel and Farmer, 2019). Piglets, on the other hand, face environmental and dietary changes that lead to issues such as diarrhea and infections by pathogenic bacteria, resulting in disruptions to gut microbiota, immunity, and metabolism (Chen et al., 2023; Chu et al., 2020; He et al., 2019; Yang et al., 2017; Yu et al., 2017).

Antibiotics were previously used to treat diarrhea caused by pathogenic bacteria in piglets. In light of the rise of multidrug-resistant strains of bacteria (Su et al., 2018; Yang et al., 2019), the European Union implemented a comprehensive prohibition on the utilization of antibiotics as growth promoters starting in 2006, and China has completely banned in-feed antibiotics since 2020 (Van Boeckel et al., 2015). Therefore, the pig industry must explore effective strategies, particularly antibiotic alternatives in feed additives, to enhance animal health.

Probiotics, the most promising antibiotic alternative, have been found effective in treating or preventing post-weaning diarrhea. Our group has focused on studying *Lactobacillus* and *Bacillus*, the most common probiotic bacteria (Chen et al., 2023; Chu et al., 2020; He et al., 2019; Shan et al., 2022; Xia et al., 2020; Yang et al., 2017, 2016, 2022; Yu et al., 2017; Zhang et al., 2019, 2018, 2017; Zhou et al., 2015). Our laboratory and others have found probiotics play a crucial role in preserving gut microbiota homeostasis, modulating immunity, and improving the growth performance of piglets (Lin et al., 2020; Yang et al., 2018). For example, *Lactobacillus rhamnosus* GG alleviates the development of intestinal inflammation in piglets infected with *Salmonella* (Yang et al., 2017; Yu et al., 2017; Zhang et al., 2018). Orally fed *Bacillus licheniformis* and *Bacillus subtilis* spore mixture (BLS-mix) alleviates *Escherichia coli* (*E. coli*)-induced diarrhea and small intestinal inflammation (Yang et al., 2016; Zhang et al., 2017; Zhou et al., 2015).

Considering that combinations of probiotics are more effective than single strains and may be more effective in expanding the spectrum of protection against microbial infections (Chapman et al., 2013), a select mixture of *Lactobacillus* and *Bacillus* was developed. We have found oral administration of CBB-mix (composed of *Lactobacillus johnsonii* [CJ21], *Bacillus subtilis* [BS15], and *Bacillus licheniformis* [BL21]) alleviates *Salmonella*-induced ileal inflammation of piglets (Liu et al., 2019). Probiotic CBB-mix can also attenuate dysbiosis of colonic microbiota and increase the abundance of butyric acid-producing bacteria in response to *Salmonella* infantis in newly weaned piglets (Chu et al., 2020). However, the effects of CBB-mix supplementation on the growth performance of piglets in clinical farms have not been studied.

In addition to directly feeding probiotics to piglets, healthy sow gut microbiota structure is important for the intestinal health of piglets due to the existence of the gut–breast axis (Bian et al., 2016). Vertical transmission via placenta and breast milk contributes to bacterial colonization, and the development of digestive and immune systems, reducing diarrhea in piglets (Prentice et al., 2016; Rodríguez et al., 2021). Breast milk contains large amounts of proteins and secretory immunoglobulins that provide specific immunity to the newborn (Kalbermatter et al., 2021). Colostrum extracts antibodies from the mother's mucosal barrier, enabling the newborn to resist the same antigens as in the mother's environment (Atyeo and Alter, 2021). Therefore, it is feasible and effective to improve the immunity and the gut microbiota structure of the offspring by altering the metabolic composition of the milk and thereby preventing dysbiosis. Recent studies have shown that probiotics can regulate sow milk metabolism through the gut–breast axis, which in turn can influence the structure of the offspring's gut microbiota and improve their immune status (Cuinat et al., 2022). However, the effects of maternal CBB-mix supplementation on the health of sows and their offspring by regulation of the gut–breast axis remain unknown.

This study investigated the effects of maternal and/or offspring supplementation with CBB-mix on the diarrhea incidence, growth performance, immunity, and fecal microbiota of piglets. The results offer theoretical guidance for clinical application of probiotic CBB-mix and indicate that maternal probiotic supplementation improves health status of sows and growth performance of offspring piglets via the gut–breast axis.

## 2 Materials and methods

### 2.1 Animal ethics statement

All animals in this study adhered strictly to the Guidelines for Laboratory Animal Use and Care established by the Chinese Center for Disease Control and Prevention, as well as the Rules for Medical Laboratory Animals (1998) issued by the Chinese Ministry of Health. The experimental protocol (AW71013202-2-1) was approval from the Animal Ethics Committee of the China Agricultural University.

### 2.2 Bacterial strains

CJ21, BS15, and BL21 were isolated from healthy pig intestinal contents and stored in our lab (Chu et al., 2020). The probiotic bacteria were prepared by the State Key Laboratory of Feeding Microbiology Engineering of Beijing Da Bei Nong Group, containing 1 × 10^12^ CFU/g of CJ21, 1 × 10^9^ CFU/g of BS15, and 1 × 10^9^ CFU/g of BL21, freeze-dried as bacterial powder and stored at −20 °C.

### 2.3 Animals, feed, and experimental design

**Fig. 1:**
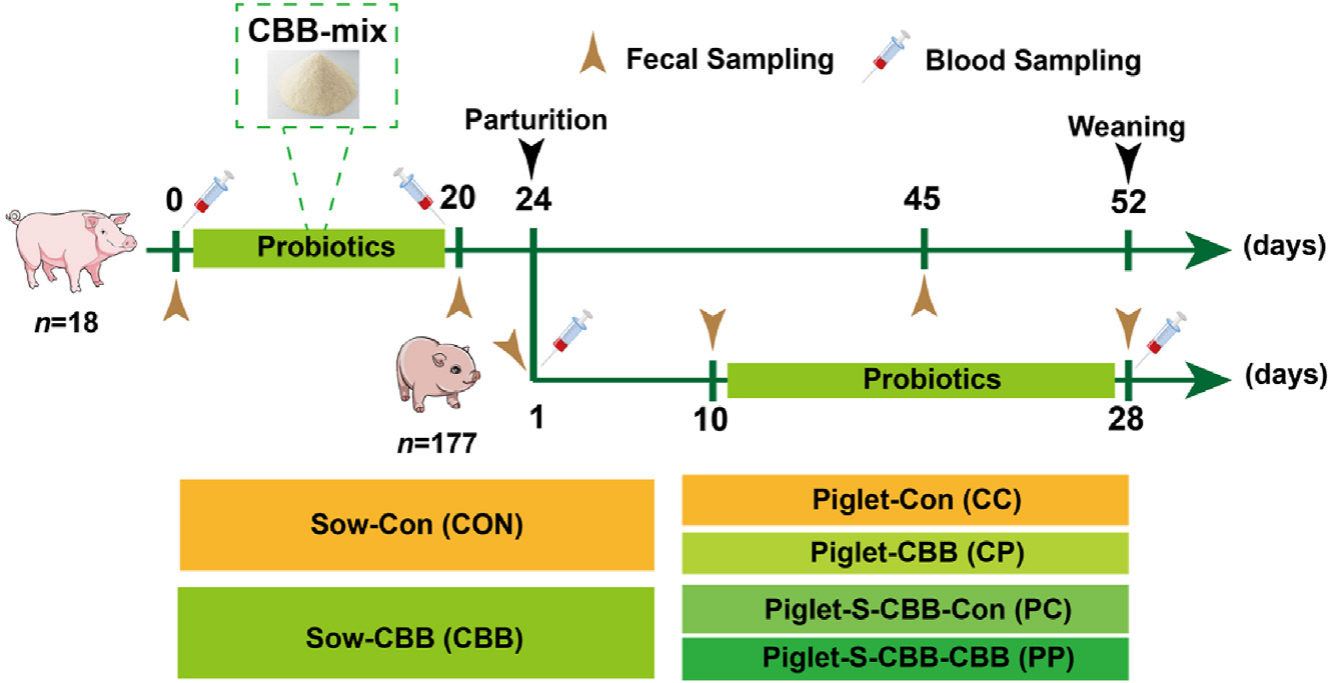
Experimental design. Sows were supplemented daily with CBB-mix from days 0 to 20. CON sows were fed a basal diet and CBB sows were fed a basal diet plus CBB-mix (containing 1×10^12^ CFU/g of *Lactobacillus johnsonii* [CJ21], 1×10^9^ CFU/g of *Bacillus subtilis* [BS15], and 1×10^9^ CFU/g of *Bacillus licheniformis* [BL21], *n* = 9). A total of 177 newborn piglets from 18 litters in the CBB and CON groups were divided into four groups at 10 days of age. The CC group, sows and their offspring piglets were fed a basal diet (*n* = 40 from 5 litters); the CP group, sows fed the basal diet and their offspring piglets fed the basal diet plus CBB-mix (*n* = 38 from 5 litters); the PC group, sows fed the basal diet plus CBB-mix and their offspring piglets fed the basal diet (*n* = 50 from 4 litters); and the PP group, both sows and their offspring piglets fed the basal diet plus CBB-mix (*n* = 49 from 5 litters).

Eighteen Yorkshire × Landrace sows of similar parity and gestational age were equally randomized into two groups and fed with or without CBB-mix for 20 days before parturition as shown in Fig. 1. The groups of sows were as follows: 1) control (CON) group (basal diet, *n* = 9), 2) CBB-mix (CBB) group (basal diet + CBB-mix, *n* = 9).

After parturition, a total of 177 suckling piglets were divided into four groups fed with or without CBB-mix in the creep feed from 11 to 28 days of age after parturition. Before feeding CBB-mix each day, the piglet feed replenishment tray was emptied and the mixed feed was added to ensure that it was completely consumed before replenishing with new feeds. The groups of piglets were as follows: 1) CC group (basal diet, *n* = 40 from 5 litters of CON sows); 2) CP group (basal diet + CBB-mix, *n* = 38 from 4 litters of CON sows); 3) PC group (basal diet, *n* = 50 from 4 litters of CBB sows); 4) PP group (basal diet + CBB-mix, *n* = 49 from 4 litters of CBB sows). Sows and piglets did not receive antibiotics during the experiment. The diets were formulated to contain equal quantities of crude protein and digestive energy (DE), meeting the nutrient requirements specified by the NRC (2012). Composition and nutrient levels of the basal diet were shown in Table 1. The contents of crude protein were determined using a Kjeldahl automated apparatus (K9805, Shanghai Analytical Instrument Co., Ltd., Shanghai, China) following the methods 976.06 (AOAC, 2007). Mineral contents, including phosphorus (P) and calcium (Ca), were analyzed using the 5110 ICP-OES (Agilent Technologies Australia (M) Pty. Ltd., Australia) following the methods 995.11 and 927.02 (AOAC, 2007). The available P was calculated according to NRC (2012). Total lysine contents were determined via HPLC (Water HPLC system, Water Corporation, MA, USA) following the methods 982.30 (AOAC, 2007). The DE was computed by dividing the DE content of the diet by the inclusion rate of grains in the diet (NRC, 2012).

**Table 1:** Composition and nutrient levels of the basal diet (air-dried basis, %).

Item	Late pregnancy of sows	Lactation of sows	Suckling piglets
**Ingredients**			
Corn	60.58	62.58	67.98
Soybean oil	3.00	3.00	2.00
Bran	5.00	6.00	24.00
Soybean meal	25.00	22.00	2.00
Steam fish meal	3.00	3.00	0.02
Phytase	0.02	0.02	0.70
Ca(HCO_3_)_2_	0.80	0.80	1.00
Mineral feed	1.10	1.10	0.30
NaCl	0.50	0.50	1.00
Vitamin-mineral premix^1^	1.00	1.00	1.00
Total	100.00	100.00	100.00
**Nutrient levels**^2^			
Digestible energy, MJ/kg	13.37	13.81	13.77
Crude protein	17.50	18.50	19.32
Calcium	0.95	0.95	0.84
Phosphorus	0.75	0.75	6.04
Available phosphorus	0.45	0.45	5.73
Total lysine	0.85	1.10	1.22

1 The vitamin-mineral premix provided the following per kilogram of diets: vitamin A 400,000 IU, vitamin D_3_ 120,000 IU, vitamin E 4500 IU, vitamin K_3_ 210 mg, vitamin B_2_ 800 mg, vitamin B_6_ 450 mg, niacinamide 3700 mg, calcium pantothenate 2000 mg, folic acid 500 mg, biotin 60 mg, choline chloride 50 mg, Cu 0.6 g, Fe 6 g, Zn 4 g, Mn 2 g, I 50 mg, Se 10 mg.

2 Digestible energy and available phosphorus were calculated values, and other nutrient levels were measured values.

Sow feces were collected on days 0, 20, and 45 (21 days after parturition), and piglet feces were obtained at 1, 10, and 28 days of age. Blood samples (5 mL) from the jugular vein of sows (*n* = 9) before farrowing and piglets (*n* = 6) from each group were obtained for serum collection. The serum was stored at −20 °C before further analysis.

### 2.4 Sample collection and assessment of diarrhea

The litter size and weight on 1 day after cross-fostering, and the litter size at weaning and litter weight at weaning, as well as the average daily gain (ADG) and piglet mortality were recorded to evaluate the sow production performance and piglet growth performance. The ADG per litter of all piglets from 1 to 28 days of age was calculated. The duration of labor and estrus interval of sows were recorded, and the 28 d total milk production was calculated (28-day total milk production = ADG × 28 × litter size × 4). The feed intake of sows after parturition were weighted and average daily feed intake (ADFI) were calculated (ADFI = total daily intake for 28 days/28). The constipation rate of sows was recorded.

Backfat thickness of sows was measured on days 0, 10, 20, 30, 40, and 52 following CBB-mix supplementation using an A8 all-digital B-ultrasound diagnostic instrument (Ruisheng Chaoying Electronic Technology Co., Ltd., Xuzhou, China). Each sow was measured 3 times at each time point.

After farrowing and cross-fostering, all neonatal piglets were weighed in 24 h and piglets weighing less than 800 g were recorded as weak piglets. All piglets underwent daily clinical examination including diarrhea assessment using fecal samples. The diarrhea score was recorded for 28 days since birth as previously described (Hu et al., 2019). Specifically, 0, columnar-shaped stool; 1, moist soft stool; 2, thick, shapeless, feces without fecal water separation; 3, liquid, shapeless, and feces with fecal water separation. A piglet with a fecal score of 3 was considered to have diarrhea and the diarrhea incidence of piglets was calculated.

The fecal score of sows after farrowing was recorded as follows (Lu et al., 2022): 0, no feces; 1, hard, compact, or grainy texture feces; 2, solid feces; 3, mildly firm feces; 4, soft, flat or very damp feces. Sows with a fecal score of 0 or 1 were recognized as constipated.

### 2.5 16S rRNA sequencing and microbiota data analysis

Total microbial genomic DNA extracted from the feces of sows and piglets was extracted using the E.Z.N.A. bacterial DNA Kit (Omega Biotek, Norcross, GA, USA) following the manufacturer's instructions. The quality and concentration of DNA were assessed and preserved at −80 °C. Amplification of the V3 to V4 hypervariable region of the bacterial 16S rRNA gene was amplified on an ABI GeneAmp 9700 PCR thermocycler (ABI, CA, USA) using primer pairs 338F (5′-ACTCCTACGGGAGGCAGCAG-3′) and 806R (5′-GGACTACHVGGGTWTCTAAT-3′) (Liu et al., 2016). Equimolar amounts of purified amplicons were pooled together and then subjected to paired-end sequencing on an Illumina MiSeq PE300 platform (Illumina, San Diego, CA, USA) from Majorbio (Shanghai, China).

Raw fastq files underwent de-multiplexing via an in-house Perl script, followed by quality-filtering using fastq version 0.19.6 (Chen et al., 2018) and merging facilitated by FLASH version 1.2.7 (Magoč and Salzberg, 2011). The refined sequences were then clustered into operational taxonomic units (OTU) utilizing UPARSE (version 7.1, http://drive5.com/uparse/) with 97% sequence similarity (Edgar, 2013). Taxonomic classification of each out-representative sequence was performed using the Ribosomal Database Project (RDP) Classifier (http://rdp.cme.msu.edu/) against the Silva v138 database, with a confidence threshold of 70%. Alpha diversity indexes (Sobs, Shannon, and Simpson indexes) were calculated using Mothur version 1.31.2 (http://www.mothur.org). Similarities between microbial communities across samples were assessed via partial least squares discriminant analysis (PLS-DA) utilizing the R package (version 3.3.1). A heatmap illustrating the relative abundance of OTU was generated using R software (version 2.15) (http://www.R-project.org).

### 2.6 Untargeted metabolomics

Sow milk underwent LC-MS/MS analysis using a Thermo UHPLC-Q Exactive HF-X system equipped with an ACQUITY HSS T3 column (100 mm × 2.1 mm i.d., 1.8 μm; Waters, USA) at Majorbio (Shanghai, China). Mass spectrometric data acquisition utilized a Thermo UHPLC-Q Exactive HF-X Mass Spectrometer with electrospray ionization (ESI) source operating in both positive and negative modes. Next, PLS-DA was performed using the R package “ropls” (Version 1.6.2). Based on the variable importance projection (VIP) obtained by the PLS-DA model and the *P*-value obtained by the student's *t*-test, metabolites with VIP > 1 and *P* < 0.05 were judged to be significantly different. Enrichment and pathway analysis for the altered metabolites based on the KEGG database (http://www.genome.jp/kegg/) were performed by Python packages (scipy.stats, https://docs.scipy.org/doc/scipy/).

### 2.7 Enzyme-linked immunosorbent assay (ELISA)

Cytokines interleukin-1β (IL-1β), interleukin-8 (IL-8), interleukin-18 (IL-18), tumor necrosis factor-α (TNF-α), immunoglobulin G (IgG), and immunoglobulin A (IgA) contents in serum were measured with ELISA kits (Jianglai, Shanghai, China except for IL-8 and IgA from Solarbio, Beijing, China). The contents of lactoferrin (LF), IgG and IgA in milk was measured with ELISA kits, following the manufacturer's instructions (Solarbio, Beijing, China).

### 2.8 Detection of serum oxidative stress markers

Serum total antioxidant capacity (T-AOC), superoxide dismutase (SOD) activity, and malondialdehyde (MDA) content were quantified by commercial kits (S0101S, S0119, and S0131S, Beyotime, Shanghai, China), in accordance with the manufacturer's instructions.

### 2.9 Serum iron measurement

Serum iron content was assessed using a commercial kit (Solarbio, Beijing, China), while serum total iron-binding capacity (TIBC) content was determined by a TIBC assay kit (Solarbio, Beijing, China), as per the manufacturer's guidelines.

### 2.10 Detection of serum myeloperoxidase (MPO) content

Serum MPO content was quantified using an assay kit according to the manufacturer's instructions (Nanjing Jiancheng Bioengineering Institute, Nanjing, China).

### 2.11 Statistical analysis

Statistical analysis was conducted using SPSS 23.0 (Chicago, IL, USA). Data are expressed as means ± standard of error mean (SEM). Comparisons between two groups were evaluated using the independent samples *t*-test. For analyses involving multiple groups, one-way ANOVA was employed, followed by Tukey's multiple comparison test. Differences in gut microbiota abundance across multiple groups were assessed using the non-parametric Kruskal–Wallis test, with FDR test for post-hoc. Correlation analysis between microbial communities and colostrum metabolome was performed using Pearson's correlation coefficient. A significance level of *P* < 0.05 was considered statistically different. ∗*P* < 0.05, ∗∗*P* < 0.01, and ∗∗∗*P* < 0.001.

## 3 Results

### 3.1 CBB-mix feeding improved the production performance of sows

As shown in Table 2, the CBB group had higher litter size at birth (*P* = 0.030) and litter weight at birth (*P* = 0.010), increasing by 25% and 19% compared with the CON group, respectively. These results indicate that maternal CBB-mix supplementation can effectively improve sow production performance.

**Table 2:** Effects of maternal dietary supplementation with CBB-mix during late pregnancy on production performance of sows.^1^

Item	CON (*n* = 9)	CBB (*n* = 9)	*P*-value
ADFI, kg	3.03 ± 0.562	3.26 ± 0.495	0.763
Constipation rate, %	0.13 ± 0.018	0.09 ± 0.012	0.175
Duration of labor, h	5.00 ± 0.577	3.87 ± 0.307	0.102
Estrus interval, day	5.78 ± 1.544	3.89 ± 0.111	0.240
Total milk production of 28 days, kg	1866.58 ± 255.381	2473.30 ± 305.035	0.113
Litter size at birth	10.22 ± 0.830^a^	12.78 ± 0.683^b^	0.030
Number of healthy birth	9.00 ± 0.799	11.11 ± 0.807	0.082
Average weight at birth, kg	1.29 ± 0.054	1.35 ± 0.057	0.147
Litter weight at birth, kg	13.74 ± 0.582^a^	16.35 ± 0.655^b^	0.010

ADFI = average daily feed intake.

^a,b^Values with different superscript lower-case letters within the same row are significantly different (*P* < 0.05).

1 Data are presented as means ± standard of error mean (SEM). CON group, sows fed a basal diet; CBB group, sows fed the basal diet plus CBB-mix (containing 1×10^12^ CFU/g of *Lactobacillus johnsonii* [CJ21], 1×10^9^ CFU/g of *Bacillus subtilis* [BS15], and 1×10^9^ CFU/g of *Bacillus licheniformis* [BL21]). Sows fed the basal diet plus CBB-mix from days 0 to 20. The first day of sows supplemented with CBB-mix is defined as day 0.

In addition, we measured the effects of CBB-mix for different durations on backfat loss in sows after farrowing, as sows need to mobilize their energy reserves during lactation to meet the nutritional needs of piglets, resulting in backfat loss (Lavery et al., 2019; Thiengpimol et al., 2022). It was found that backfat loss in CON sows was up to 1.32 mm from days 20 to 52 after delivery, while backfat increased by 0.58 mm in the CBB group (Table 3). Backfat thickness in the CBB group on day 40 (*P* = 0.025) and 52 (*P* = 0.017) was significantly higher than that of the CON group (Table 3). In addition, CBB-mix supplementation in late gestation doubled backfat growth in sows on day 20 compared to the CON group. The results showed that feeding CBB-mix effectively reduced backfat loss in sows after farrowing.

**Table 3:** Effects of adding CBB-mix in late gestation on backfat of sows.^1^

Item	CON (*n* = 9)	CBB (*n* = 9)	*P*-value
Backfat thickness, mm			
Day 0 (20 days before farrowing)	14.69 ± 0.719	15.02 ± 0.675	0.744
Day 10	15.24 ± 0.751	15.57 ± 0.723	0.756
Day 20 (4 days before farrowing)	15.36 ± 0.999	16.23 ± 0.510	0.446
Day 30	16.23 ± 0.760	16.83 ± 0.479	0.512
Day 40	15.24 ± 0.768^a^	17.77 ± 0.673^b^	0.025
Day 52 (pre-weaning)	14.04 ± 0.527^a^	16.81 ± 0.900^b^	0.017
Backfat growth (days 20 to 0), mm	0.66 ± 0.558	1.21 ± 0.481	0.468
Backfat loss (days 20 to 52), mm	1.32 ± 0.956	−0.58 ± 0.909	0.170

^a,b^Values with different superscript lower-case letters within the same row are significantly different (*P* < 0.05).

1 Data are presented as means ± standard of error mean (SEM). CON group, sows fed a basal diet; CBB group, sows fed the basal diet plus CBB-mix (containing 1×10^12^ CFU/g of *Lactobacillus johnsonii* [CJ21], 1×10^9^ CFU/g of *Bacillus subtilis* [BS15], and 1×10^9^ CFU/g of *Bacillus licheniformis* [BL21]). The first day of sows supplemented with CBB-mix is defined as day 0.

### 3.2 Maternal CBB-mix supplementation improved the growth performance of piglets

The effects of CBB-mix on growth performance and diarrhea incidence in piglets are shown in Table 4 and Table 5, respectively. Piglets in the PC and PP groups showed significantly increased litter size at weaning compared with the CC group (*P* < 0.001), indicating maternal CBB-mix supplementation improved offspring growth.

**Table 4:** Comparison of maternal and offspring supplementation of CBB-mix on growth performance of suckling piglets.^1^

Item	CC	CP	PC	PP	*P-*value
Litter size at weaning	7.50 ± 0.500^a^	8.33 ± 0.333^ab^	12.50 ± 0.289^b^	9.80 ± 0.735^b^	<0.001
Piglet mortality, %	0.19 ± 0.083	0.14 ± 0.068	0.12 ± 0.047	0.19 ± 0.049	0.713
Average weight at weaning, kg	6.93 ± 0.442	7.29 ± 1.180	6.65 ± 0.273	6.49 ± 0.346	0.825
Litter weight at weaning, kg	60.86 ± 9.655	67.09 ± 10.877	81.74 ± 3.371	61.75 ± 6.684	0.275
ADFI, g	56.97 ± 14.312	73.96 ± 21.007	80.84 ± 25.258	69.61 ± 19.649	0.876
ADG, kg	1.79 ± 0.213	1.87 ± 0.362	2.29 ± 0.121	1.67 ± 0.197	0.329

ADFI = average daily feed intake; ADG = average daily gain.

^a,b^Values with different superscript lower-case letters within the same row are significantly different (*P* < 0.05).

1 Data are presented as means ± standard of error mean (SEM). CC group, piglets and sows fed a basal diet (*n* = 40 from 5 litters); CP group, piglets fed the basal diet plus CBB-mix (containing 1×10^12^ CFU/g of *Lactobacillus johnsonii* [CJ21], 1×10^9^ CFU/g of *Bacillus subtilis* [BS15], and 1×10^9^ CFU/g of *Bacillus licheniformis* [BL21]) and sows fed basal diet (*n* = 38 from 4 litters); PC group, piglets fed a basal diet and sows fed the basal diet plus CBB-mix (*n* = 50 from 4 litters); PP group, both piglets and sows fed the basal diet plus CBB-mix (*n* = 49 from 5 litters).

**Table 5:** Comparison of maternal and offspring supplementation of CBB-mix on diarrhea incidence of suckling piglets.^1^

Item	At risk^2^	With diarrhea^3^		*P*-value		
	*n*	*n*	Diarrhea incidence,%			
**Before CBB-mix (1 to 10 days of age)**				CBB		
CON group (*n* = 90)	848	64	7.55	0.007		
CBB group (*n* = 117)	1085	50	4.61			
**After CBB-mix (11 to 28 days of age)**				CP	PC	PP
CC group (*n* = 42)	744	10	1.34	0.012	0.025	0.999
CP group (*n* = 40)	698	1	0.14		0.638	0.005
PC group (*n* = 51)	910	3	0.33			0.011
PP group (*n* = 53)	894	13	1.45			
**Total (1 to 28 days of age)**				CP	PC	PP
CC group (*n* = 40)	1187	58	4.89	<0.001	<0.001	0.061
CP group (*n* = 38)	1103	17	1.54		0.380	0.034
PC group (*n* = 50)	1431	16	1.12			<0.001
PP group (*n* = 49)	1458	50	3.43			

1 CON group, the offspring piglets of sows fed a basal diet; CBB group, the offspring piglets of sows fed the basal diet plus CBB-mix; CC group, piglets and sows fed a basal diet (*n* = 40 from 5 litters); CP group, piglets fed the basal diet plus CBB-mix (containing 1×10^12^ CFU/g of *Lactobacillus johnsonii* [CJ21], 1×10^9^ CFU/g of *Bacillus subtilis* [BS15], and 1×10^9^ CFU/g of *Bacillus licheniformis* [BL21]) and sows fed a basal diet (*n* = 38 from 4 litters); PC group, piglets fed a basal diet and sows fed the basal diet plus CBB-mix (*n* = 50 from 4 litters); PP group, piglets and sows fed the basal diet plus CBB-mix (*n* = 49 from 5 litters). Sows fed the basal diet plus CBB-mix from days 0 to 20. Piglets of the CP and PP group fed the basal diet plus CBB-mix from 11 to 28 days of age.

2 The number of at risk indicates the total number of piglets per day for a period of time.

3 The sum of the number of piglets with diarrhea during this period is considered to be with diarrhea.

Furthermore, the diarrhea incidence of piglets was analyzed. Compared with CON offspring, the diarrhea incidence of piglets in the CBB group was significantly lower before 10 days of age (*P* = 0.007). After 10 days of age, piglets in the CP and PP groups began CBB-mix supplementation. From days 11 to 28 or from days 1 to 28, there was a significant decrease in diarrhea incidence for CP and PC groups versus CC and PP groups (*P* < 0.05). These results indicate maternal CBB-mix supplementation reduces offspring diarrhea and similar effects are achieved by feeding CBB-mix to suckling piglets.

### 3.3 Effects of CBB-mix on systemic immunity, iron metabolism, and antioxidant capacity in sows

Due to childbirth stress, sows are more susceptible to immune deficiency and inflammation at delivery. Results showed serum IgG (*P* = 0.020) and IgA (*P* < 0.001) contents in the CBB group on day 20 were significantly higher compared with day 1, and also increased compared with the CON group on day 20 (*P* = 0.030, *P* = 0.011, Table 6). The serum TNF-α (*P* < 0.001) and IL-1β (*P* = 0.006) contents of sows in the CON group increased on day 20 compared with day 1 (Table 6). After CBB-mix treatment, the contents of pro-inflammatory TNF-α and IL-1β in serum on day 20 were maintained at a similar level to day 1 (*P* > 0.05) and significantly lower than the CON group on day 20 (*P* = 0.001 and *P* = 0.036, respectively, Table 6). The serum IL-8 content of the CBB group on day 20 was significantly lower than that of the CON group (*P* = 0.022, Table 6). However, serum MPO and IL-18 contents were not affected by CBB-mix (*P* > 0.05, Table 6).

**Table 6:** Effects of maternal CBB-mix supplementation during late pregnancy on the immune function, iron metabolism, inflammatory factors, antioxidant capacity, and milk lactoferrin and immunoglobulins of sows.^1^

Item	CON	CBB	*P*-value
**IgG, mg/mL**			
Day 1	1.17 ± 0.155	1.22 ± 0.225^A^	0.965
Day 20	1.31 ± 0.133^a^	1.96 ± 0.149^bB^	0.030
*P*-value	0.768	0.020	
**IgA, μg/mL**			
Day 1	0.58 ± 0.255^A^	2.13 ± 2.260^A^	0.282
Day 20	4.15 ± 2.377^aB^	8.06 ± 4.363^bB^	0.011
*P*-value	0.007	<0.001	
**Serum iron, μmol/L**			
Day 1	8.32 ± 1.110^bB^	4.33 ± 1.000^a^	0.001
Day 20	5.85 ± 2.317^A^	6.43 ± 0.556	0.807
*P*-value	0.032	0.070	
**TIBC, μmol/L**			
Day 1	1223.84 ± 20.994	1262.77 ± 62.430	0.661
Day 20	1100.74 ± 59.646^a^	1250.34 ± 21.828^b^	0.042
*P*-value	0.081	0.964	
**TNF-α, pg/mL**			
Day 1	34.48 ± 1.701^A^	36.99 ± 6.605	0.911
Day 20	69.58 ± 6.824^bB^	40.37 ± 2.561^a^	0.001
*P*-value	<0.001	0.822	
**IL-1β, pg/mL**			
Day 1	35.49 ± 1.947^A^	35.61 ± 5.347	0.999
Day 20	50.08 ± 3.805^bB^	37.21 ± 1.189^a^	0.036
*P*-value	0.006	0.951	
**IL-18, pg/mL**			
Day 1	20.04 ± 2.572	18.59 ± 3.603	0.924
Day 20	20.96 ± 1.485	17.63 ± 3.684	0.684
*P*-value	0.968	0.968	
**IL-8, pg/mL**			
Day 1	332.89 ± 39.453	299.47 ± 59.439^B^	0.828
Day 20	271.88 ± 35.332^b^	96.14 ± 15.674^aA^	0.022
*P*-value	0.506	0.015	
**T-AOC, mmol/L**			
Day 1	1.164 ± 0.045	1.218 ± 0.044	0.773
Day 20	1.060 ± 0.074	1.224 ± 0.068	0.167
*P*-value	0.400	0.997	
**SOD, U/mL**			
Day 1	0.44 ± 0.018^a^	0.50 ± 0.063^b^	0.010
Day 20	0.72 ± 0.044^a^	0.87 ± 0.067^b^	0.004
*P*-value	0.846	0.125	
**MDA, μmol/L**			
Day 1	329.31 ± 58.222	328.33 ± 70.040	0.999
Day 20	310.70 ± 67.311	207.62 ± 26.805	0.404
*P*-value	0.967	0.297	
**MPO, U/L**			
Day 1	161.26 ± 21.120	147.33 ± 5.679	0.796
Day 20	151.96 ± 18.107	115.82 ± 5.573	0.302
*P*-value	0.902	0.391	
**Milk lactoferrin, pg/mL**			
Day 1 after parturition	44.35 ± 8.461	75.25 ± 26.455	0.840
Day 7 after parturition	44.98 ± 10.280^a^	236.45 ± 88.058^b^	0.008
Day 21 after parturition	60.48 ± 14.552	79.78 ± 44.584	0.980
*P*-value	0.553	0.103	
**Milk IgG, mg/mL**			
Day 1 after parturition	0.79 ± 0.057	1.31 ± 0.195	0.385
Day 7 after parturition	1.58 ± 0.411	1.81 ± 0.340	0.929
Day 21 after parturition	1.49 ± 0.296	1.63 ± 0.272	0.977
*P*-value	0.117	0.443	
**Milk IgA, μg/mL**			
Day 1 after parturition	2.09 ± 0.782^aB^	6.83 ± 2.704^bB^	0.003
Day 7 after parturition	0.21 ± 0.026^A^	0.36 ± 0.156^A^	0.999
Day 21 after parturition	0.29 ± 0.063^A^	0.18 ± 0.023^A^	0.999
*P*-value	0.011	0.005	

IgG = immunoglobulin G; IgA = immunoglobulin A; TIBC = total iron-binding capacity; TNF-α = tumor necrosis factor-α; IL-1β = interleukin-1β; IL-18 = interleukin-18; IL-8 = interleukin-8; T-AOC = total antioxidant capacity; SOD = superoxide dismutase; MDA = malondialdehyde; MPO = myeloperoxidase.

^a, b^Different superscipts in the same row indicate significant differences between groups on the same day (*P* < 0.05). ^A, B^Different superscipts in the same column indicate significant differences between different time with in the same group (*P* < 0.05).

1 Data are presented as means ± standard of error mean (SEM) (*n* = 6). CON group, sows fed a basal diet; CBB group, sows fed the basal diet plus CBB-mix (containing 1×10^12^ CFU/g of *Lactobacillus johnsonii* [CJ21], 1×10^9^ CFU/g of *Bacillus subtilis* [BS15], and 1×10^9^ CFU/g of *Bacillus licheniformis* [BL21]). The first day of sows supplemented with CBB-mix was defined as day 0.

It was found that serum iron content in the CON group decreased significantly on day 20 compared to day 1 (*P* = 0.032), indicating that iron deficiency occurred in gestating sows before farrowing over time. CBB-mix had a tendency to mitigate this decline (*P* = 0.070, Table 6). We also examined the effects of CBB-mix on the antioxidant capacity of sows and found that CBB-mix-fed sows had increased serum antioxidant SOD activity on day 20 (*P* = 0.004, Table 6).

The IgA content in sow colostrum was significantly increased in the CBB group compared with the CON group on day 1 after parturition (*P* = 0.003, Table 6). Together, these results indicate CBB-mix effectively improves sows' immune response, antioxidant capacity, and iron metabolism.

### 3.4 Effects of maternal CBB-mix supplementation on systemic immunity, iron metabolism, and antioxidant capacity in piglets

Compared with the CC, CP, and PC piglets, the PP piglets had higher serum IgA content at 1 day of age (*P* > 0.05) and the PP group had a higher IgG content at 28 days of age compared with 1 day of age (*P* = 0.012, Table 7). It was found that TNF-α contents in piglets at 28 days of age were significantly decreased compared with 1 day of age across all four groups (*P* < 0.001, Table 7). Feeding CBB-mix to both sows and their offsprings (PP group) had the tendency of reducing the IL-8 content at 28 days of age compared with the CC group (*P* = 0.073), but the contents of IL-18 and IL-1β showed no significant changes among four groups (*P* > 0.05).

**Table 7:** Effects of maternal and/or offspring dietary supplementation with CBB-mix on immune function, iron metabolism, inflammatory factors, and antioxidant capacity in of piglets.^1^

Item	CC	CP	PC	PP	*P*-value
**IgG, mg/mL**					
1 day of age	2.55 ± 0.326	2.55 ± 0.326	2.52 ± 0.614	2.95 ± 0.122^A^	0.722
28 days of age	3.67 ± 0.282	3.59 ± 0.547	3.49 ± 0.326	4.44 ± 0.097^B^	0.269
*P*-value	0.084	0.147	0.317	0.012	
**IgA, μg/mL**					
1 day of age	5.34 ± 1.885	5.67 ± 2.593^B^	4.48 ± 0.844	12.91 ± 3.446^B^	0.053
28 days of age	0.37 ± 0.092	0.26 ± 0.035^A^	0.29 ± 0.035	0.71 ± 0.509^A^	0.613
*P*-value	0.054	0.049	0.156	<0.001	
**Serum iron, μmol/L**					
1 day of age	3.60 ± 0.214^a^	3.38 ± 0.189^aA^	6.92 ± 0.512^bd^	6.92 ± 0.582^cd^	<0.001
28 days of age	5.41 ± 0.483	8.90 ± 1.324^B^	9.04 ± 0.440	9.37 ± 0.798	0.065
*P*-value	0.310	<0.001	0.230	0.074	
**TIBC, μmol/L**					
1 day of age	416.17 ± 40.918	458.45 ± 76.928^A^	431.73 ± 473.962^A^	348.80 ± 28.247^A^	0.436
28 days of age	662.26 ± 174.230^a^	1,118.42 ± 87.455^bcB^	1,255.36 ± 11.866^cB^	968.10 ± 58.976^abcB^	0.015
*P*-value	0.119	<0.001	<0.001	<0.001	
**TNF-α, pg/mL**					
1 day of age	130.33 ± 5.874^B^	139.04 ± 6.904^B^	136.97 ± 11.578^B^	131.37 ± 12.411^B^	0.909
28 days of age	60.11 ± 6.247^A^	44.39 ± 2.517^A^	57.99 ± 1.403^A^	43.15 ± 7.661^A^	0.183
*P*-value	<0.001	<0.001	<0.001	<0.001	
**IL-1β, pg/mL**					
1 day of age	31.00 ± 3.415	29.16 ± 2.173	28.18 ± 2.491	31.62 ± 2.719	0.860
28 days of age	30.34 ± 2.236	28.11 ± 1.161	23.47 ± 1.756	28.56 ± 1.339	0.126
*P*-value	0.999	0.997	0.699	0.916	
**IL-18, pg/mL**					
1 day of age	21.43 ± 1.104	20.61 ± 1.613	23.45 ± 4.049	21.81 ± 1.354	0.789
28 days of age	25.43 ± 1.256	19.86 ± 0.833	20.43 ± 0.215	24.92 ± 0.691	0.470
*P*-value	0.073	0.997	0.752	0.588	
**IL-8, pg/mL**					
1 day of age	341.63 ± 15.078	330.31 ± 14.087	299.89 ± 12.794	287.46 ± 30.197	0.214
28 days of age	365.58 ± 54.673	278.78 ± 10.105	237.81 ± 29.507	182.46 ± 51.028	0.073
*P*-value	0.973	0.747	0.469	0.072	
**T-AOC, mmol/L**					
1 day of age	1.05 ± 0.066	1.02 ± 0.112	1.28 ± 0.095^B^	1.22 ± 0.070^B^	0.172
28 days of age	0.84 ± 0.067^a^	1.27 ± 0.045^b^	0.97 ± 0.078^a^^A^	0.86 ± 0.037^a^^A^	0.004
*P*-value	0.173	0.152	0.036	0.021	
**SOD, U/mL**					
1 day of age	0.12 ± 0.033^a^	0.12 ± 0.033^a^	0.72 ± 0.071^bB^	0.29 ± 0.046^a^	<0.001
28 days of age	0.10 ± 0.044^a^	0.12 ± 0.012^a^	0.38 ± 0.070^bA^	0.44 ± 0.041^b^	<0.001
*P*-value	0.993	0.999	<0.001	0.090	
**MDA, μmol/L**					
1 day of age	110.95 ± 19.418	69.45 ± 15.885^A^	88.28 ± 4.167^A^	83.62 ± 24.604^A^	0.449
28 days of age	243.12 ± 39.392^a^	408.96 ± 33.031^bB^	237.83 ± 27.608^aB^	456.15 ± 51.105^bB^	0.004
*P*-value	0.073	<0.001	0.035	<0.001	
**MPO, U/L**					
1 day of age	309.41 ± 49.456	251.72 ± 11.240^A^	247.79 ± 60.550	265.49 ± 21.632	0.649
28 days of age	482.20 ± 36.275^b^	605.70 ± 39.593^bB^	298.92 ± 89.373^a^	252.51 ± 45.655^a^	0.002
*P*-value	0.052	<0.001	0.928	0.999	

IgG = immunoglobulin G; IgA = immunoglobulin A; TIBC = total iron-binding capacity; TNF-α = tumor necrosis factor-α; IL-1β = interleukin-1β; IL-18 = interleukin-18; IL-8 = interleukin-8; T-AOC = total antioxidant capacity; SOD = superoxide dismutase; MDA = malondialdehyde; MPO = myeloperoxidase.

^a^^-^^c^Different superscripts in the same row indicate significant differences between groups on the same day (*P* < 0.05). ^A, B^Different superscripts in the same column indicate significant differences between different times with in the same group (*P* < 0.05).

1 Data are presented as means ± standard of error mean (SEM) (*n* = 6). CC group, sows and their offspring piglets fed a basal diet; CP group, sows fed a basal diet and their offspring piglets fed the basal diet plus CBB-mix (containing 1×10^12^ CFU/g of *Lactobacillus johnsonii* [CJ21], 1×10^9^ CFU/g of *Bacillus subtilis* [BS15], and 1×10^9^ CFU/g of *Bacillus licheniformis* [BL21]) ; PC group, sows fed a basal diet plus CBB-mix and their offspring piglets fed a basal diet; PP group, both sows and their offspring piglets fed the basal diet plus CBB-mix. Sows fed the basal diet plus CBB-mix from days 1 to 20. Piglets of the CP and PP groups were fed basal diet plus CBB-mix from 11 to 28 days of age postnatally.

The effects of maternal and offspring CBB-mix supplementation on iron metabolism is shown in Table 7. Maternal CBB-mix supplementation in late gestation significantly increased the serum iron content in their offspring piglets (PC and PP groups) at 1 day of age compared with the CC and CP groups, respectively (*P* < 0.05). However, there was no significant difference in serum TIBC content among the four groups at 1 day of age (*P* > 0.05). At 28 days of age, compared to the CC group, CBB-mix supplementation to either sows or their offsprings had the tendency to increase the serum iron content in the CP, PC, and PP groups (*P* = 0.065), while the TIBC contents of piglets in the CP and PC groups were significantly increased compared with CC group (*P* = 0.015). In addition, compared to 1 day of age, the serum iron content of the CP group was significantly higher at 28 days of age (*P* < 0.001), and the TIBC contents of the CP, PC, and PP groups were also significantly higher at 28 days of age compared to 1 day of age (*P* < 0.001). These data indicate that maternal CBB-mix supplementation improves the iron homeostasis of the piglets.

Additionally, the antioxidant capacity of piglets at birth (1 day of age) and before weaning (28 days of age) was examined (Table 7). Maternal CBB-mix supplementation in late gestation significantly increased offspring serum SOD activity at 1 day of age (PC and PP groups) compared to the non-supplemented CC and CP groups (*P* < 0.001, Table 7). The SOD activity in the PP group was significantly higher than in the CC and CP groups at 28 days of age (*P* < 0.001). Continuous CBB-mix feeding to piglets (CP group) also significantly increased serum T-AOC at 28 days of age versus CC, PC, and PP groups (*P* = 0.004). These data indicate that both maternal and offspring CBB-mix supplementation improves the antioxidant capacity of suckling piglets.

At 28 days of age, serum MPO content in the PC and PP groups significantly decreased compared with the CC group (Table 7), while MPO content in the CP group was higher than the PC and PP groups at 28 days of age (*P* < 0.05), and significantly higher than that at 1 day of age (*P* < 0.001). This indicates that CBB-mix feeding to both piglets and sows resulted in significantly lower serum MPO content in piglets compared to other groups. Collectively, the aforementioned results suggest that maternal CBB-mix supplementation benefits piglet systemic immunity, iron metabolic homeostasis, and antioxidant capacity.

### 3.5 Oral administration of CBB-mix optimized the gut microbiota in sows

**Fig. 2:**
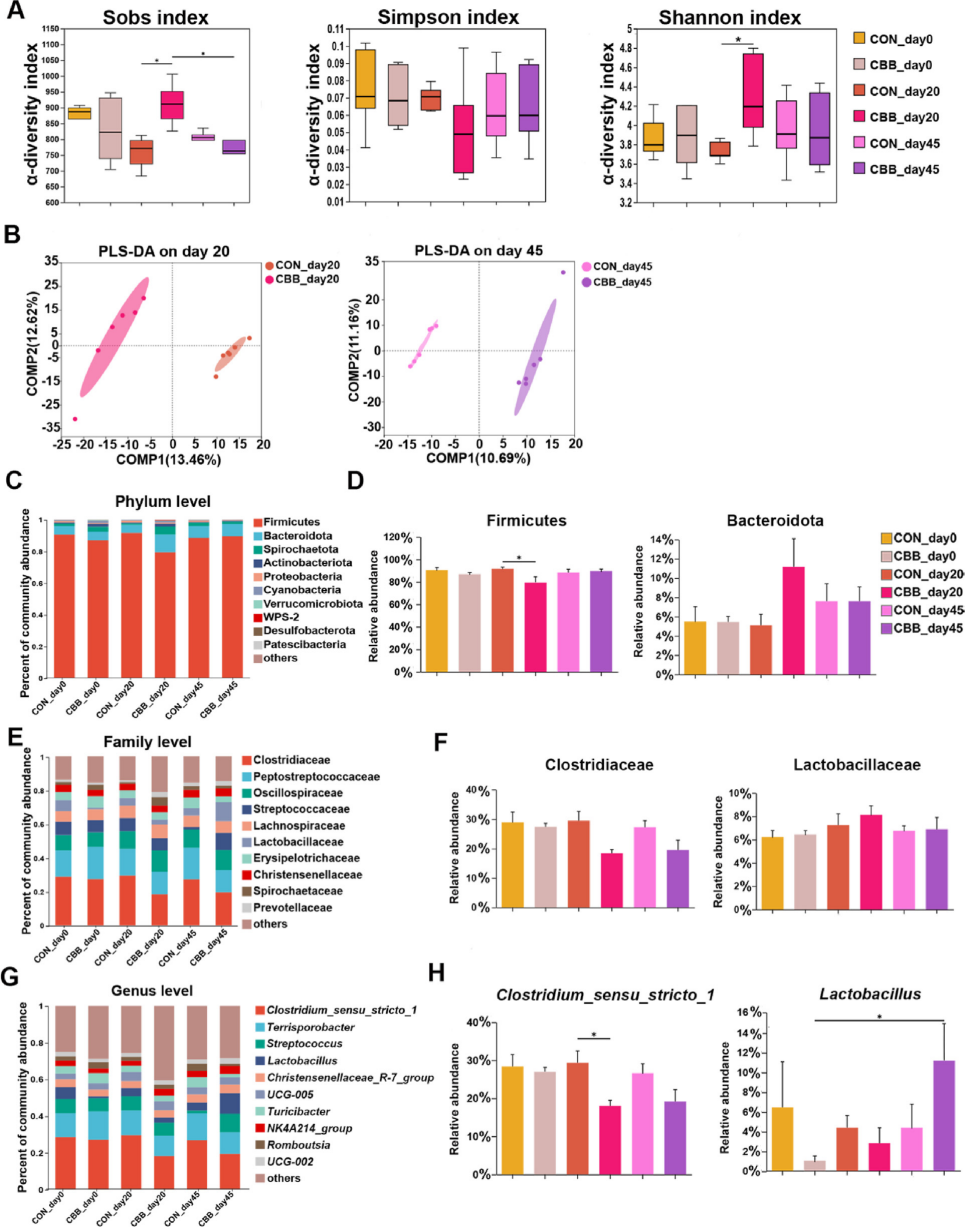
CBB-mix optimized gut microbiota in sows. (A) Fecal bacterial α-diversity indexes (Sobs, Simpson, and Shannon indexes). (B) Partial least squares discriminant analysis (PLS-DA) cluster analysis of fecal microbiota. Colored ellipses indicate 0.95 confidence interval (CI) ranges within each group. (C) The relative abundance of fecal bacteria at the phylum level. (D) The differences in the relative abundance of Firmicutes and Bacteroidota among each group. (E) The relative abundance of fecal bacteria at the family level. (F) The differences in the relative abundance of Clostridiaceae and Lactobacillaceae among groups. (G) The relative abundance of fecal bacteria at the genus level. (H) The differences in the relative abundance of *Clostridium_sensu_stricto_1* and *Lactobacillus* among groups. COMP = components. The first day of sows supplemented with CBB-mix is defined as day 0. CON_day0, the feces of sows fed a basal diet on day 0; CBB_day0, the feces of sows fed the basal diet plus CBB-mix (containing 1×10^12^ CFU/g of *Lactobacillus johnsonii* [CJ21], 1×10^9^ CFU/g of *Bacillus subtilis* [BS15], and 1×10^9^ CFU/g of *Bacillus licheniformis* [BL21]) on day 0; CON_day20, the feces of sows fed a basal diet on day 20; CBB_day20, the feces of sows fed the basal diet plus CBB-mix on day 20; CON_day45, the feces of sows fed a basal diet on day 45; CBB_day45, the feces of sows fed the basal diet plus CBB-mix on day 45. Data are presented as means ± standard of error mean (SEM) (*n* = 6). ∗, *P* < 0.05.

Compared to the CON group, the CBB group exhibited increased community richness and diversity (by Sobs, Shannon, and Simpson indexes) on day 20 (Fig. 2A). Upon cessation of CBB-mix feeding, OTU decreased rapidly to control levels by day 45 (Fig. 2A). PLS-DA plots showed distinct clusters between the CBB group's fecal microbiota from CON on days 20 and 45 (Fig. 2B). Across all samples, 17 phyla were identified (Fig. S1A); Firmicutes and Bacteroidetes dominated (Fig. 2C). Compared with the CON group, the CBB group exhibited decreased relative abundance of Firmicutes on day 20 (*P* = 0.046, Fig. 2D). At the genus level, *Clostridium_sensu_stricto_1*, *Terrisporobacter*, *Streptococcus*, *Lactobacillus* and *Christensenellaceae_R-7_group* were the top 5 abundant taxa (Fig. 2G). CBB-mix increased the relative abundance of *Lactobacillus* on day 45 versus day 0 (*P* = 0.041), while decreasing the relative abundance of *Clostridium_sensu_stricto_1* on day 20 compared with the CON group (*P* = 0.027, Fig. 2H).

Furthermore, the correlation between fecal microbiota and phenotypic data in sows was explored. Multiple relative abundance of *Lachnospiraceaes* exhibited positive correlations with serum T-AOC and IgG content, and negative correlations with IL-1β content (*P* < 0.05, Fig. S1B). Other short-chain fatty acid-producers, including the relative abundance of *Romboutsia* and *Eubacterium-coprostanoligenes_group*, displayed positive correlations with serum MPO content and negative correlations with MDA and IL-18 contents (*P* < 0.05, Fig. S1B). These data indicate CBB-mix optimizes gut microbiota by fostering the growth of beneficial microbes and suppressing pathogens.

### 3.6 CBB-mix altered the sow milk metabolome

**Fig. 3:**
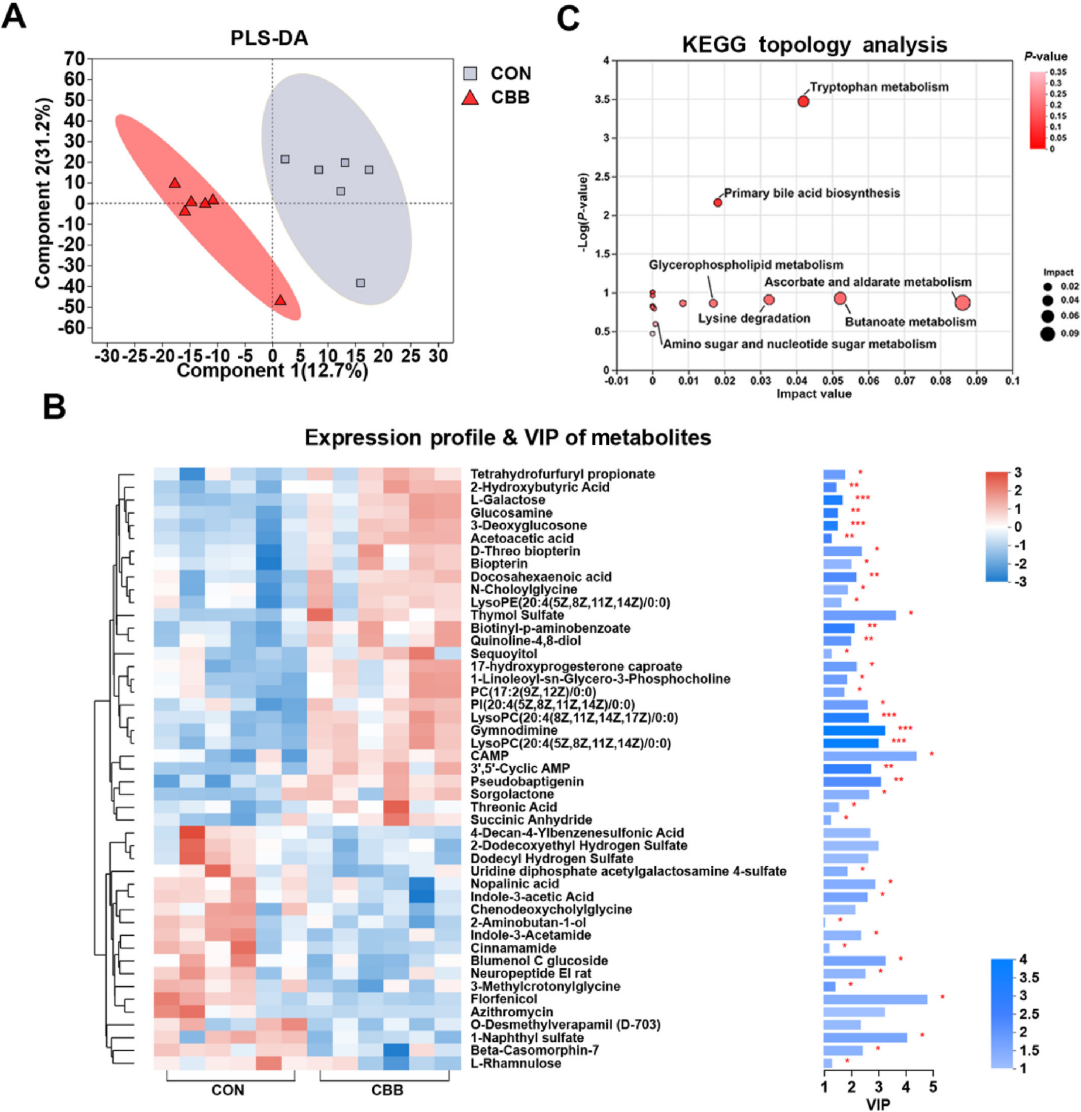
Effects of CBB-mix on the milk metabolome of sows. (A) Partial least squares discriminant analysis (PLS-DA) plot of maternal milk metabolites (ANOSIM analysis, *P* < 0.001). (B) Heatmap of differential metabolites [variable importance projection (VIP) > 1.0, *P* < 0.05]. (C) Metabolic pathways for altered milk metabolites based on the KEGG database. CON group, sows fed a basal diet; CBB group, sows fed the basal diet plus CBB-mix (containing 1×10^12^ CFU/g of *Lactobacillus johnsonii* [CJ21], 1×10^9^ CFU/g of *Bacillus subtilis* [BS15], and 1×10^9^ CFU/g of *Bacillus licheniformis* [BL21]). *n* = 6. ∗, *P* < 0.05; ∗∗, *P* < 0.01; ∗∗∗, *P* < 0.001.

Colostrum is the most important source of nutrition and immunocompetence for newborn piglets. The PLS-DA plot of colostrum metabolomes showed that clusters between CBB and CON groups were clearly separated (Fig. 3A). A total of 43 metabolites exhibited differential levels between CBB and CON sows (VIP > 1.0, *P* < 0.05, Fig. 3B). Notably, CBB-mix significantly increased concentrations of 16 compounds, including 17-hydroxyprogesterone caproate, gymnodimine, docosahexaenoic acid, N-choloylglycine, and pseudobaptigenin in milk, while significantly reducing concentrations of 14 compounds, including beta-casomorphin-7, chenodeoxycholylglycine, indole-3-acetic acid, and indole-3-acetamide (VIP > 1.0, *P* < 0.05, Fig. 3B). KEGG pathway enrichment analysis revealed that CBB-mix-altered metabolites were involved in tryptophan metabolism (indole-3-acetamide, indole-3-acetic acid, and quinoline-4,8-diol), primary bile acid biosynthesis (chenodeoxycholylglycine and N-choloylglycine), ascorbate and aldarate metabolism (L-galactose), and butanoate metabolism (acetoacetic acid) (*P* < 0.05, Fig. 3C).

Then, the correlation analysis between colostrum differential metabolites, serum parameters of piglets at 1 day of age, and growth performance indices of piglets at 28 days of age were performed (Fig. S2). It was found that indole-3-acetic acid content exhibited a negative correlation with serum T-AOC and IgG and iron contents. Quinoline-4,8-diol content displayed a positive correlation with serum iron content. N-choloylglycine content showed a negative correlation with serum IL-8 content but a positive correlation with serum iron content. L-galactose content demonstrated positive correlations with serum T-AOC, IgG, and iron content, but a negative correlation with IL-1β production. Acetoacetic acid content was positively correlated with serum iron content and T-AOC but negatively correlated with serum IL-8 content. Diarrhea incidence was negatively correlated with ADG, litter weight at weaning, and serum TIBC content (*P* < 0.05, Fig. S2). These results suggest CBB-mix influences sow milk metabolism by regulating tryptophan and bile acid metabolism via the gut microbiota–breast axis.

### 3.7 Maternal CBB-mix supplementation contributed to homeostasis of the gut microbiota in the offspring

**Fig. 4:**
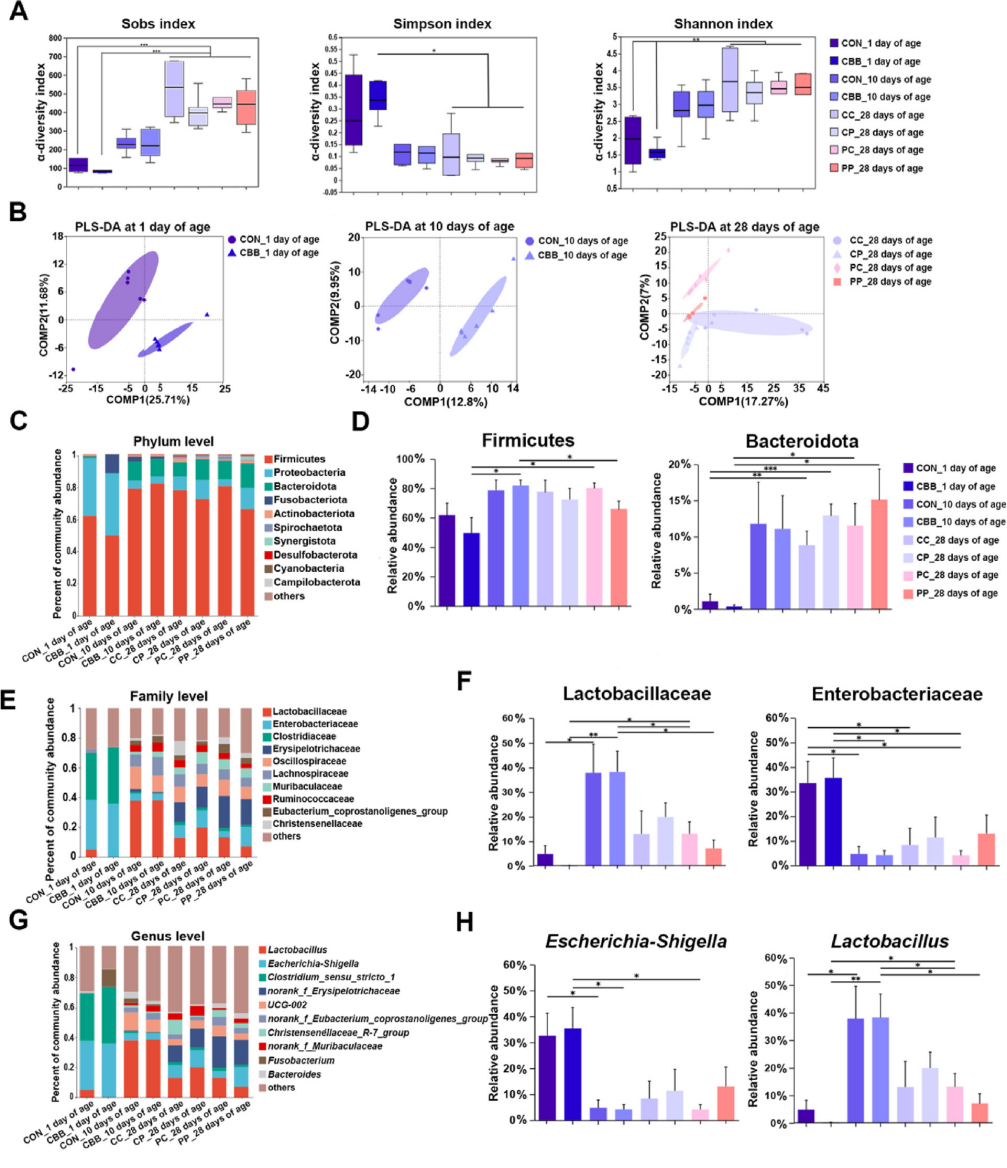
CBB-mix improved the gut microbiota homeostasis in piglets. (A) Fecal bacterial α-diversity indexes (Sobs, Simpson, and Shannon indexes). (B) Partial least squares discriminant analysis (PLS-DA) cluster analysis of fecal microbiota. (C) The relative abundance of fecal bacteria at the phylum level in piglets. (D) The differences in the relative abundance of Firmicutes and Bacteroidota among groups. (E) The relative abundance of fecal bacteria at the family level in piglets. (F) The differences in the relative abundance of Lactobacillaceae and Enterobacteriaceae among groups. (G) The relative abundance of fecal bacteria at the genus level in piglets. (H) The differences in the relative abundance of *Escherichia-Shigella* and *Lactobacillus* among groups. COMP = components. The birth date of piglets was recorded as day 0. CON_1 day of age, the feces of the offspring piglets with sows fed a basal diet at 1 day of age; CBB_1 day of age, the feces of the offspring piglets with sows fed the basal diet plus CBB-mix (containing 1×10^12^ CFU/g of *Lactobacillus johnsonii* [CJ21], 1×10^9^ CFU/g of *Bacillus subtilis* [BS15], and 1×10^9^ CFU/g of *Bacillus licheniformis* [BL21]) at 1 day of age; CON_10 days of age, the feces of the offspring piglets with sows fed a basal diet at 10 days of age; CBB_10 days of age, the feces of the offspring piglets with sows fed the basal diet plus CBB-mix at 10 days of age; CC_28 days of age, the feces of the offspring piglets with sows and piglets fed a basal diet at 28 days of age; CP_28 days of age, the feces of the offspring piglets with sows fed the basal diet plus CBB-mix and piglets fed basal diet at 28 days of age; PC_28 days of age, the feces of the offspring piglets with sows fed a basal diet and piglets fed the basal diet plus CBB-mix at 28 days of age; PP_28 days of age, the feces of the offspring piglets with sows and piglets fed the basal diet plus CBB-mix at 28 days of age. Data are presented as means ± standard of error mean (SEM) (*n* = 6). ∗, *P* < 0.05; ∗∗, *P* < 0.01; ∗∗∗, *P* < 0.001.

Furtherly, the fecal microbiota at different ages of piglets from sows with or without CBB-mix supplementation were investigated. Compared with 1 day of age, a noticeable increase in community richness and diversity (as indicated by Sobs, Shannon, and Simpson indexes) was observed across all four groups at 28 days of age (Fig. 4A). PLS-DA plots showed distinct clusters between groups at 1, 10, and 28 days of age (Fig. 4B). Among 20 phyla identified (Fig. S3A), Firmicutes and Proteobacteria were the most abundant bacteria (Fig. 4C). Compared with the CBB group at 1 day of age, a higher relative abundance of Firmicutes was observed for the CBB group at 10 days of age and PC group at 28 days of age, whereas a lower relative abundance of Firmicutes was observed in the PP group at 28 days of age compared with the CBB group at 10 days of age (*P* < 0.05, Fig. 4D). Additionally, the relative abundance of Bacteroidetes was significantly increased in piglets at 28 days of age compared with piglets at 1 day of age, indicating age effects on growth or survival of gut Bacteroidetes before weaning (*P* < 0.05, Fig. 4D).

At family level, compared with 1 day of age, maternal CBB-mix supplementation led to increased fecal Lactobacillaceae and decreased Enterobacteriaceae relative abundance of the offspring at 10 days of age (*P* < 0.05, Fig. 4F). Consistent with the above results, at genus level, maternal CBB-mix supplementation increased fecal *Lactobacillus* relative abundance in offspring at 10 days of age, while decreasing *Escherichia-Shigella* relative abundance at 10 and 28 days of age (*P* < 0.05, Fig. 4G and H). As expected, direct feeding of CBB-mix to piglets increased the relative abundance of fecal *Lactobacillus* but decreased *Escherichia-Shigella* in CP group at 28 days of age compared to the CON group at 1 day of age (*P* < 0.05, Fig. 4H).

Correlation analysis of piglet fecal microbiota and serum parameters at 1 day of age revealed a negative correlation between the relative abundance of *Lactobacillus* and serum MDA content, while the relative abundance of *Escherichia-Shigella* exhibited a positive correlation with serum IgA content. Additionally, the relative abundance of *Clostridium_sensus_stricto_1* was positively correlated with serum TNF-α content, and the relative abundance of *Bacteroides* showed positive correlations with serum IL-18 content, while having negative correlations with litter weight at weaning and survival rate (*P* < 0.05, Fig. S3B).

As we can see from Fig. S3B, the correlation analysis of fecal microbiota and serum parameters from piglets at 28 days of age revealed a positive correlation between the relative abundance of *Escherichia-Shigella* and ADG, whereas the relative abundance of *Clostridium_sensu_strico_1* exhibited a negative correlation with ADG. The relative abundance of Erysipelotrichaceae showed a positive correlation with serum TIBC content and the relative abundance of *Eubacterium-coprostanoligenes_group* was positively correlated with serum IgA content. The relative abundance of *Christensenellaceae_R-7_group* exhibited positive correlations with serum TNF-α and IgA contents, but negative correlations with serum TIBC content. The relative abundance of Muribaculaceae displayed a positive correlation with serum IL-1β content but negative correlations with serum SOD activity, litter weight at weaning, and survival rate. The relative abundance of *Bacteroides* demonstrated positive correlations with serum SOD activity and iron content, and negative correlations with serum MPO content.

### 3.8 Sow gut microbiota influenced colostrum metabolites and offspring gut microbiota structure

**Fig. 5:**
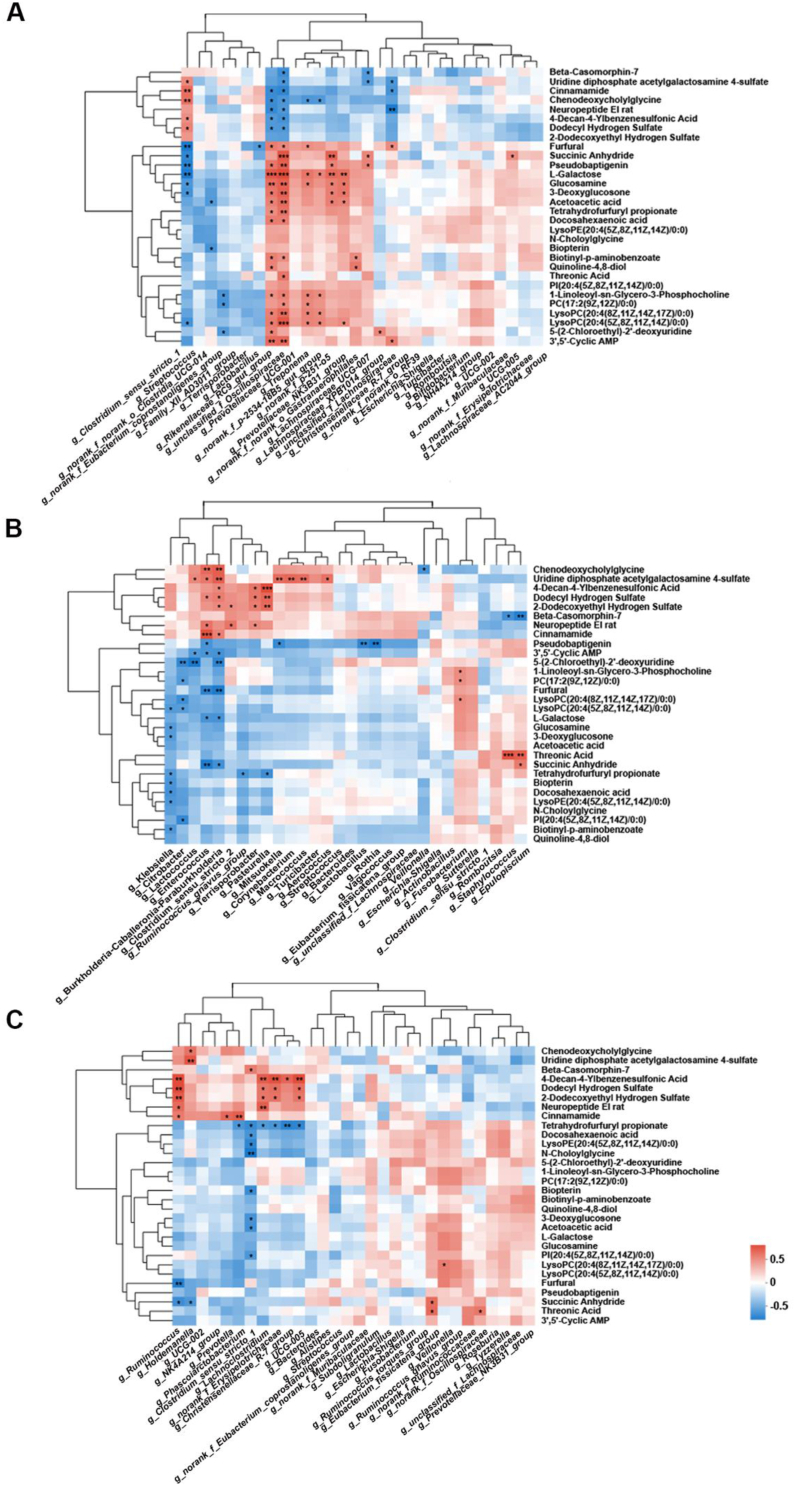
Correlation analysis of fecal microbiota and sow colostrum metabolome. (A) Pearson's correlations between milk metabolites and sow fecal microbiota on day 20. (B) Pearson's correlations between milk metabolites and piglet fecal microbiota at 1 day of age. (C) Pearson's correlations between milk metabolites and piglet fecal microbiota at 10 days of age. *n* = 6. ∗, *P* < 0.05; ∗∗, *P* < 0.01; ∗∗∗, *P* < 0.001.

To investigate the relationship between fecal microbiota and colostrum metabolome, the association analyses were performed, which involving fecal bacteria at the genus level from both sows and piglets, correlated with sow colostrum metabolites (Fig. 5). It was shown that fecal the relative abundance of Lachnospiraceae, *Prevotellaceae_NK3B31_group*, *p-251-o5*, *p-2534-18B5_gut_group*, *Oscillospiraceae* (*P* < 0.05), *Rikenellaceae_RC9_gut_group*, *Treponema* (*P* < 0.05), and *Clostridium_sensu_stricto_1* in sows on day 20 exhibited significant correlations with more than 5 metabolites (Fig. 5A), suggesting important roles in influencing the sow's colostrum metabolome.

Correlation analysis between piglet fecal microbiota (1 day of age) and colostrum metabolome showed that the relative abundance of *Fusobacterium*, *Lactococcus*, *Klebsiella*, *Pasteurella*, *Terrisporobacter*, *Burkholderia-Caballeronia-Paraburkholderia*, *Citrobacter*, *Enterococcus*, and *Epulopiscium* were correlated with more than 3 metabolites (*P* < 0.05, Fig. 5B).

Correlation analysis between piglet fecal microbiota (10 days of age) and colostrum metabolome showed that the relative abundance of *Lachnoclostridium*, *Ruminococcus*, *UCG-005*, Erysipelotrichaceae, and *Clostridium_sensu_stricto_1* were correlated with more than 3 metabolites (*P* < 0.05, Fig. 5C). These results indicate alterations in the gut microbiota of sows before farrowing affect colostrum metabolome and thus piglet gut microbiota.

## 4 Discussion

The gut microbiota plays a pivotal role in pig health, with homeostatic gut microbiota providing beneficial host effects (Zhu et al., 2022). Probiotics can regulate fecal microflora structure and immune response in pigs (Kim and Isaacson, 2015), while maternal supplementation benefits offspring by shaping gut microbiota structure and improving growth performance (Han et al., 2022; Kalbermatter et al., 2021; Nissen et al., 2022; Zhu et al., 2022). Here, feeding CBB-mix to sows in late gestation optimized their intestinal microbiota structure, influencing colostrum metabolome and consequently enhancing the growth performance and immunity of the offspring. Direct CBB-mix administration to lactating piglets also improved growth performance and gut dysbiosis.

Controlling maternal diet is an effective strategy to bolster offspring health, particularly in terms of gut health, via gut microbiota (Wang et al., 2022a, 2021). Maternal probiotic mixture intake not only improves the reproductive performance of sows but also reduces diarrhea incidence of offspring and fosters the growth performance of piglets (Betancur et al., 2021; Han et al., 2022; Lan and Kim, 2020). Consistent with these studies, our study indicates maternal CBB-mix supplementation in late pregnancy improved production performance of sows and growth performance of offspring, and alleviated diarrhea occurrence in suckling piglets.

‘Gut closure’ occurs in piglets within 36 h post-birth. Therefore, piglets consumption of antibody-rich colostrum is necessary within these 36 h to facilitate maternal passive protection, optimal iron metabolism, immunity, and antioxidant capacity. Colostrum contains substances that influence thermoregulation, growth, organ development, and immune system maturation in piglets (Zhou et al., 2019). This study showed pregnant sows had iron dyshomeostasis and inflammation to a certain degree pre-parturition, whereas maternal CBB-mix supplementation increased sow and piglet serum iron content and SOD activity. Additionally, LF and immunoglobulin in milk during lactation were increased by CBB-mix. Consistent with this, probiotics enhance immune responses to enteric infection and improve piglet growth by increasing antimicrobial proteins and LF contents in milk (Donovan et al., 2021) and inhibiting pro-inflammatory cytokines (Dhanani and Bagchi, 2013; Xin et al., 2020). These results suggest a positive impact of CBB-mix on offspring. As CBB-mix increases immunoglobulin and LF contents in milk, the primary nutritional source for newborn piglets, this positive vertical effect may be sustained via the gut–breast axis.

Gut development in offspring is profoundly affected by the maternal gut microbiota and breast milk (Yang et al., 2021). Probiotic supplementation during pregnancy alters the structure of the maternal gut microbiota, thereby beneficially affecting their offspring (Cuinat et al., 2022; Zhang et al., 2020). However, this effect depends on the probiotic species administered and the timing of administration (Cuinat et al., 2022). Maternal CBB-mix supplementation increased the diversity of fecal bacteria in offspring at both 10 and 28 days of age. Notably, maternal CBB-mix supplementation significantly increased *Lactobacillus* relative abundance but decreased *Escherichia-Shigella* relative abundance in offspring at 10 days of age. The results strongly indicate CBB-mix probiotic properties can be transmitted from sows to their offspring via the gut–breast axis.

The main ways mothers transmit their gut microbiota to offspring include 1) vertical transmission through the uterus pre-delivery (Stout et al., 2013); 2) intrapartum transmission through the birth canal (Rutayisire et al., 2016; Wampach et al., 2017); and 3) postnatal transmission via breast milk (De Leoz et al., 2015; Jost et al., 2015). Efficiency of absorption and metabolism in the maternal gastrointestinal tract significantly affects the composition of breast milk (Rodríguez et al., 2021). Moreover, probiotics significantly increase colostrum protein and milk fat content (Han et al., 2022), and milk LF boosts the immune response and promotes anti-inflammatory responses to pathogenic bacteria (Donovan, 2016). In humans, breast milk promotes the production of aromatic lactic acid by *Bifidobacterium* in the infant gut, potentially affecting early-life immune function (Laursen et al., 2021). Thus, it was speculate feeding CBB-mix to sows modulates the piglet gut microbiota by increasing beneficial bacteria like *Lactobacillus* via the gut–breast axis.

*Lactobacillus* plays an important role in host trophic and metabolic functions, protecting the host (Han et al., 2022). In our study, *Lactobacillus* was one of the main dominant bacteria in the gut microbiota of sows and piglets. Moreover, maternal CBB-mix supplementation significantly increased *Lactobacillus* relative abundance in offspring at 10 days of age. This suggests piglets vertically inherit some dominant bacteria from sows. Maternal CBB-mix supplementation balances gut microbiota of sows, and this probiotic trait is vertically transmitted to offspring improving their gut health.

In addition, CBB-mix may affect piglet gut microbiota by altering milk metabolism. Maternal gut microbiota can metabolize many food components, and metabolites produced can travel through the bloodstream to the breast, then to the infant's gut through the blood-milk barrier (Gay et al., 2018). A probiotic-containing fermented diet increases amino acid, organic acid, D-glutamine, and D-glutamate metabolism in sow milk (Wang et al., 2022a). In this study, tryptophan metabolism [indole-3-acetamide, indole-3-acetic acid (IAA), and quinoline-4,8-diol] and primary bile acid biosynthesis (chenodeoxycholylglycine and N-choloylglycine) were affected by CBB-mix.

Notably, colostrum quinoline-4,8-diol and N-choloylglycine content was increased, while indole-3-acetamide, IAA, and chenodeoxycholylglycine contents were decreased in CBB-mix-fed sows. Bacterial tryptophan metabolites, including indole and indole acid derivatives, are potent bioactive metabolites that exert anti-inflammatory effects in the gut by activating the pregnane X receptor or the aryl hydrocarbon receptor (AhR) (Gao et al., 2018). Indole-3-acetic acid is an important indole-derivative that has been shown to have local anti-inflammatory and antioxidant effects (Roager and Licht, 2018). *Bacteroides thetaiotaomicron* effectively alleviates colitis by increasing levels of IAA and its ligand AhR (Li et al., 2021).

In addition, bile acids play a variety of roles in the intestinal flora, such as glucose regulation and intestinal motility (Huang et al., 2019). Chenodeoxycholylglycine (glycochenodeoxycholic acid) is a hydrophobic bile acid formed in the liver by deoxycholic acid and glycine (Wang et al., 2022b). In this study, chenodeoxycholylglycine was decreased in the milk of the CBB group. Further, we found that chenodeoxycholylglycine was negatively associated with decreased relative abundance of *Rikenellaceae_RC9_gut_group* and Oscillospiraceae of sows on day 20. The relative abundance of *Rikenellaceae_RC9_gut_group* was significantly reduced in animals with intestinal mucositis and malnutrition (Cai et al., 2021), and Oscillospiraceae was associated with reduced inflammation (Aindelis et al., 2023; Hu et al., 2022). Collectively, CBB-mix may modulate the structure of the gut microbiota of piglets by altering the intestinal microbiota of sows, thereby impacting the milk metabolome, particularly in tryptophan and bile acid metabolism. However, the detailed mechanism by which CBB-mix indirectly affects piglet gut microbiota through milk metabolism still needs further investigation.

## 5 Conclusion

In conclusion, these results showed that feeding CBB-mix to pregnant sows improved gut microbiota homeostasis, production performance, and influenced colostrumtryptophan metabolism and primary bile acids biosynthesis. The altered milk metabolism is associated with improved offspring immunity and gut microbiota, ultimately reducing diarrhea incidence in suckling piglets. Additionally, CBB-mix supplementation in suckling piglets and/or maternal probiotic supplementation increased beneficial bacteria and decreased pathogenic bacteria in piglet intestines. This study provides valuable insights for CBB-mix application in the pig industry.

## Credit author statement

**Ting Hu** and **Yaohong Zhu:** Data curation, Formal analysis, Investigation, Methodology, Visualization, Writing - Original draft. **Zhiguan Song, Lan Yang, Keyuan Chen, Yi Wu,** and **Fei Xie:** Validation. **Jiufeng Wang, Guiyan Yang,** and **Yaohong Zhu:** Conceptualization, Funding acquisition, Project administration, Writing - Review & Editing. We declare that all authors have read and agreed with the manuscript.

## Declaration of competing interest

We declare that we have no financial and personal relationships with other people or organizations that can inappropriately influence our work, and there is no professional or other personal interest of any nature or kind in any product, service and/or company that could be construed as influencing the content of this paper.

## References

[bib1] Aindelis G., Ypsilantis P., Chlichlia K. (2023). Alterations in faecal microbiota and elevated levels of intestinal IgA following oral administration of *Lacticaseibacillus casei* in mice. Probiotics Antimicrob Proteins.

[bib2] AOAC International (2007).

[bib3] Atyeo C., Alter G. (2021). The multifaceted roles of breast milk antibodies. Cell.

[bib4] Betancur C., Martínez Y., Tellez-Isaias G., Castillo R., Ding X. (2021). Effect of oral administration with *Lactobacillus plantarum* CAM6 strain on sows during gestation-lactation and the derived impact on their progeny performance. Mediators Inflamm.

[bib5] Bian G., Ma S., Zhu Z., Su Y., Zoetendal E.G., Mackie R. (2016). Age, introduction of solid feed and weaning are more important determinants of gut bacterial succession in piglets than breed and nursing mother as revealed by a reciprocal cross-fostering model. Environ Microbiol.

[bib6] Cai B., Pan J., Chen H., Chen X., Ye Z., Yuan H. (2021). Oyster polysaccharides ameliorate intestinal mucositis and improve metabolism in 5-fluorouracil-treated S180 tumour-bearing mice. Carbohydr Polym.

[bib7] Chapman C.M.C., Gibson G.R., Todd S., Rowland I. (2013). Comparative in vitro inhibition of urinary tract pathogens by single- and multi-strain probiotics. Eur J Nutr.

[bib8] Chen K.Y., Wang J., Guo L., Wang J.F., Yang L., Hu T. (2023). *Lactobacillus johnsonii* L531 ameliorates *Salmonella enterica* serovar Typhimurium diarrhea by modulating iron homeostasis and oxidative stress via the IRP2 pathway. Nutrients.

[bib9] Chen S., Zhou Y., Chen Y., Gu J. (2018). Fastp: an ultra-fast all-in-one FASTQ preprocessor. Bioinformatics.

[bib10] Chu B.X., Zhu Y.H., Su J.H., Xia B., Zou Y.J., Nie J.W. (2020). Butyrate-mediated autophagy inhibition limits cytosolic *Salmonella* infantis replication in the colon of pigs treated with a mixture of *Lactobacillus* and *Bacillus*. Vet Res.

[bib11] Cuinat C., Stinson S.E., Ward W.E., Comelli Ea-O (2022). Maternal intake of probiotics to program offspring health. Curr Nutr Rep.

[bib12] De Leoz M.L.A., Kalanetra K.M., Bokulich N.A., Strum J.S., Underwood M.A., German J.B. (2015). Human milk glycomics and gut microbial genomics in infant feces show a correlation between human milk oligosaccharides and gut microbiota: a proof-of-concept study. J Proteome Res.

[bib13] Dhanani A.S., Bagchi T. (2013). *Lactobacillus plantarum* CS24.2 prevents *Escherichia coli* adhesion to HT-29 cells and also down-regulates enteropathogen-induced tumor necrosis factor-α and interleukin-8 expression. Microbiol Immunol.

[bib14] Donovan B., Suarez-Trujillo A., Casey T., Aryal U.K., Conklin D., Williams L.L. (2021). Inclusion of oat and yeast culture in sow gestational and lactational diets alters immune and antimicrobial associated proteins in milk. Animals.

[bib15] Donovan S.M. (2016). The role of lactoferrin in gastrointestinal and immune development and function: a preclinical perspective. J Pediatr.

[bib16] Edgar R.C. (2013). Uparse: highly accurate otu sequences from microbial amplicon reads. Nat Methods.

[bib17] Gao J., Xu K., Liu H.N., Liu G., Bai M.M., Peng C. (2018). Impact of the gut microbiota on intestinal immunity mediated by tryptophan metabolism. Front Cell Infect Microbiol.

[bib18] Gay M.C.L., Koleva P.T., Slupsky C.M., Toit E.D., Eggesbo M., Johnson C.C. (2018). Worldwide variation in human milk metabolome: indicators of breast physiology and maternal lifestyle?. Nutrients.

[bib20] Han L., Azad M.A.K., Huang P., Wang W., Zhang W.M., Blachier F. (2022). Maternal supplementation with different probiotic mixture from late pregnancy to day 21 postpartum: consequences for litter size, plasma and colostrum parameters, and fecal microbiota and metabolites in sows. Front Vet Sci.

[bib21] He T., Zhu Y.H., Yu J., Xia B., Liu X., Yang G.Y. (2019). *Lactobacillus johnsonii* L531 reduces pathogen load and helps maintain short-chain fatty acid levels in the intestines of pigs challenged with *Salmonella enterica* infantis. Vet Microbiol.

[bib22] Hu P., Zhao F.Z., Zhu W.Y., Wang J. (2019). Effects of early-life lactoferrin intervention on growth performance, small intestinal function and gut microbiota in suckling piglets. Food Funct.

[bib23] Hu Q., Wu C.Y., Yu J.T., Luo J.M., Peng X.C. (2022). Angelica sinensis polysaccharide improves rheumatoid arthritis by modifying the expression of intestinal Cldn5, Slit3 and Rgs18 through gut microbiota. Int J Biol Macromol.

[bib24] Huang F.J., Zheng X.J., Ma X.H., Jiang R.Q., Zhou W.Y., Zhou S.P. (2019). Theabrownin from Pu-erh tea attenuates hypercholesterolemia via modulation of gut microbiota and bile acid metabolism. Nat Commun.

[bib26] Jost T., Lacroix C., Braegger C., Chassard C. (2015). Impact of human milk bacteria and oligosaccharides on neonatal gut microbiota establishment and gut health. Nutr Rev.

[bib27] Kalbermatter C., Fernandez Trigo N., Christensen S., Ganal-Vonarburg S.C. (2021). Maternal microbiota, early life colonization and breast milk drive immune development in the newborn. Front Immunol.

[bib28] Kim H.B., Isaacson R.E. (2015). The pig gut microbial diversity: understanding the pig gut microbial ecology through the next generation high throughput sequencing. Vet Microbiol.

[bib29] Lan R., Kim I. (2020). *Enterococcus faecium* supplementation in sows during gestation and lactation improves the performance of sucking piglets. Vet Med Sci.

[bib30] Laursen M.F., Sakanaka M., Von Burg N., Mörbe U., Andersen D., Moll J.M. (2021). *Bifidobacterium* species associated with breastfeeding produce aromatic lactic acids in the infant gut. Nat Microbiol.

[bib31] Lavery A., Lawlor P.G., Magowan E., Miller H.M., O’driscoll K., Berry D.P. (2019). An association analysis of sow parity, live-weight and back-fat depth as indicators of sow productivity. Animal.

[bib32] Li K., Hao Z., Du J., Gao Y., Yang S., Zhou Y. (2021). *Bacteroides thetaiotaomicron* relieves colon inflammation by activating aryl hydrocarbon receptor and modulating CD4^+^ T cell homeostasis. Int Immunopharmacol.

[bib33] Lin B.S., Yan J.B., Zhong Z.L., Zheng X.T. (2020). A study on the preparation of microbial and nonstarch polysaccharide enzyme synergistic fermented maize cob feed and its feeding efficiency in finishing pigs. BioMed Res Int.

[bib34] Liu C.S., Zhao D.F., Ma W.J., Guo Y.D., Wang A.J., Wang Q.L. (2016). Denitrifying sulfide removal process on high-salinity wastewaters in the presence of *Halomonas* sp. Appl Microbiol Biotechnol.

[bib35] Liu X., Xia B., He T., Li D., Su J.H., Guo L. (2019). Oral administration of a select mixture of *Lactobacillus* and *Bacillus* alleviates inflammation and maintains mucosal barrier integrity in the ileum of pigs challenged with *Salmonella* infantis. Microorganisms.

[bib36] Lu D.D., Pi Y., Ye H., Wu Y.J., Bai Y., Lian S. (2022). Consumption of dietary fiber with different physicochemical properties during late pregnancy alters the gut microbiota and relieves constipation in sow model. Nutrients.

[bib37] Magoč T., Salzberg S.L. (2011). Flash: fast length adjustment of short reads to improve genome assemblies. Bioinformatics.

[bib38] National Research Council (2012).

[bib39] Nissen L., Aniballi C., Casciano F., Elmi A., Ventrella D., Zannoni A. (2022). Maternal amoxicillin affects piglets colon microbiota: microbial ecology and metabolomics in a gut model. Appl Microbiol Biotechnol.

[bib40] Prentice P., Ong K.K., Schoemaker M.H., van Tol E.A., Vervoort J., Hughes I.A. (2016). Breast milk nutrient content and infancy growth. Acta Paediatr.

[bib41] Quesnel H., Farmer C. (2019). Review: Nutritional and endocrine control of colostrogenesis in swine. Animal.

[bib42] Roager H.M., Licht T.R. (2018). Microbial tryptophan catabolites in health and disease. Nat Commun.

[bib43] Rodríguez J.M., Fernández L., Verhasselt V. (2021). The gut‒breast axis: programming health for life. Nutrients.

[bib44] Rutayisire E., Huang K., Liu Y., Tao F. (2016). The mode of delivery affects the diversity and colonization pattern of the gut microbiota during the first year of infants' life: a systematic review. BMC Gastroenterol.

[bib45] Shan Q., Liu N., Wang X., Zhu Y.H., Yin J.H., Wang J.F. (2022). *Lactobacillus* rhamnosus GR-1 attenuates foodborne *Bacillus* cereus-induced NLRP3 inflammasome activity in bovine mammary epithelial cells by protecting intercellular tight junctions. J Anim Sci Biotechnol.

[bib46] Stout M.J., Conlon B., Landeau M., Lee I., Bower C., Zhao Q. (2013). Identification of intracellular bacteria in the basal plate of the human placenta in term and preterm gestations. Am J Obstet Gynecol.

[bib47] Su J.H., Zhu Y.H., Ren T.Y., Guo L., Yang G.Y., Jiao L.G. (2018). Distribution and antimicrobial resistance of *Salmonella* isolated from pigs with diarrhea in China. Microorganisms.

[bib48] Thiengpimol P., Koonawootrittriron S., Suwanasopee T. (2022). Genetic and phenotypic correlations between backfat thickness and weight at 28 weeks of age, and reproductive performance in primiparous landrace sows raised under tropical conditions. Trop Anim Health Prod.

[bib49] Van Boeckel T.P., Brower C., Gilbert M., Grenfell B.T., Levin S.A., Robinson T.P. (2015). Global trends in antimicrobial use in food animals. Proc Natl Acad Sci U S A.

[bib50] Wampach L., Heintz-Buschart A., Hogan A., Muller E.E.L., Narayanasamy S., Laczny C.C. (2017). Colonization and succession within the human gut microbiome by archaea, bacteria, and microeukaryotes during the first year of life. Front Microbiol.

[bib51] Wang C., Wei S.Y., Liu B.J., Wang F.Q., Lu Z.Q., Jin M.L. (2022). Maternal consumption of a fermented diet protects offspring against intestinal inflammation by regulating the gut microbiota. Gut Microbes.

[bib52] Wang C., Wei S.Y., Xu B.C., Hao L.H., Su W.F., Jin M.L. (2021). *Bacillus subtilis* and *Enterococcus faecium* co-fermented feed regulates lactating sow's performance, immune status and gut microbiota. Microb Biotechnol.

[bib53] Wang L.J., Zhao G.P., Wang X.F., Liu X.X., Li Y.X., Qiu L.L. (2022). Glycochenodeoxycholate affects iron homeostasis via up-regulating hepcidin expression. Nutrients.

[bib54] Xia B., Yu J., He T., Liu X., Su J.H., Wang M.L. (2020). *Lactobacillus johnsonii* L531 ameliorates enteritis via elimination of damaged mitochondria and suppression of SQSTM1-dependent mitophagy in a *Salmonella* infantis model of piglet diarrhea. FASEB J.

[bib55] Xin J.G., Zeng D., Wang H.S., Sun N., Zhao Y., Dan Y. (2020). Probiotic *Lactobacillus johnsonii* BS15 promotes growth performance, intestinal immunity, and gut microbiota in piglets. Probiotics Antimicrob Proteins.

[bib56] Yang B., Ding M.F., Chen Y.Q., Han F.Z., Yang C.Y., Zhao J.X. (2021). Development of gut microbiota and bifidobacterial communities of neonates in the first 6 weeks and their inheritance from mother. Gut Microbes.

[bib57] Yang G.Y., Guo L., Su J.H., Zhu Y.H., Jiao L.G., Wang J.F. (2019). Frequency of diarrheagenic virulence genes and characteristics in *Escherichia coli* isolates from pigs with diarrhea in China. Microorganisms.

[bib58] Yang G.Y., Yu J., Su J.H., Jiao L.G., Liu X., Zhu Y.H. (2017). Oral administration of *Lactobacillus rhamnosus* GG ameliorates *Salmonella* infantis-induced inflammation in a pig model via activation of the IL-22bp/IL-22/STAT3 pathway. Front Cell Infect Microbiol.

[bib59] Yang G.Y., Zhu Y.H., Zhang W., Zhou D., Zhai C.C., Wang J.F. (2016). Influence of orally fed a select mixture of *Bacillus* probiotics on intestinal T-cell migration in weaned MUC4 resistant pigs following *Escherichia coli* challenge. Vet Res.

[bib60] Yang J.J., Qian K., Wang C.L., Wu Y.J. (2018). Roles of probiotic *Lactobacilli* inclusion in helping piglets establish healthy intestinal inter-environment for pathogen defense. Probiotics Antimicrob Proteins.

[bib61] Yang L., Wang J.F., Liu N., Wang X., Wang J., Yang G.H. (2022). *Lactobacillus johnsonii* L531 protects against *Salmonella* infantis-induced intestinal damage by regulating the NOD activation, endoplasmic reticulum stress, and autophagy. Int J Mol Sci.

[bib62] Yu J., Zhu Y.H., Yang G.Y., Zhang W., Zhou D., Su J.H. (2017). Anti-inflammatory capacity of *Lactobacillus rhamnosus* GG in monophasic variant *Salmonella* infected piglets is correlated with impeding NLRP6-mediated host inflammatory responses. Vet Microbiol.

[bib63] Zhang Q.Q., Li J., Cao M., Li Y., Zhuo Y., Fang Z.F. (2020). Dietary supplementation of *Bacillus subtilis* PB6 improves sow reproductive performance and reduces piglet birth intervals. Anim Nutr.

[bib64] Zhang W., Wu Q., Zhu Y.H., Yang G.Y., Yu J., Wang J.F. (2019). Probiotic *Lactobacillus rhamnosus* GG induces alterations in ileal microbiota with associated CD3-CD19^-^T-bet^+^IFNγ^+/-^ cell subset homeostasis in pigs challenged with *Salmonella enterica* serovar 4,[5],12:I. Front Microbiol.

[bib65] Zhang W., Zhu Y.H., Yang G.Y., Liu X., Xia B., Hu X. (2018). *Lactobacillus rhamnosus* GG affects microbiota and suppresses autophagy in the intestines of pigs challenged with *Salmonella* infantis. Front Microbiol.

[bib66] Zhang W., Zhu Y.H., Zhou D., Wu Q., Song D., Dicksved J. (2017). Oral administration of a select mixture of *Bacillus* probiotics affects the gut microbiota and goblet cell function following *Escherichia coli* challenge in newly weaned pigs of genotype MUC4 that are supposed to be Enterotoxigenic *E. coli* F4ab/ac receptor negative. Appl Environ Microbiol.

[bib67] Zhou D., Zhu Y.H., Zhang W., Wang M.L., Fan W.Y., Song D. (2015). Oral administration of a select mixture of *Bacillus* probiotics generates Tr1 cells in weaned F4ab/acR^−^ pigs challenged with an F4^+^ ETEC/VTEC/EPEC strain. Vet Res.

[bib69] Zhou J., Xiong X., Yin J., Zou L.J., Wang K.X., Shao Y.R. (2019). Dietary lysozyme alters sow's gut microbiota, serum immunity and milk metabolite profile. Front Microbiol.

[bib70] Zhu Q., Song M.T., Azad M.A.K., Cheng Y.T., Liu Y.T., Liu Y. (2022). Probiotics or synbiotics addition to sows' diets alters colonic microbiome composition and metabolome profiles of offspring pigs. Front Microbiol.

