# Supplementary material for: Maternal probiotic mixture supplementation optimizes the gut microbiota structure of offspring piglets through the gut–breast axis
Source: Anim Nutr. 2024 Jul 17;19:386–400. doi: 10.1016/j.aninu.2024.04.025 (PMC11617873; doi:10.1016/j.aninu.2024.04.025)
Supplement: Multimedia component 1 [file mmc1.docx]

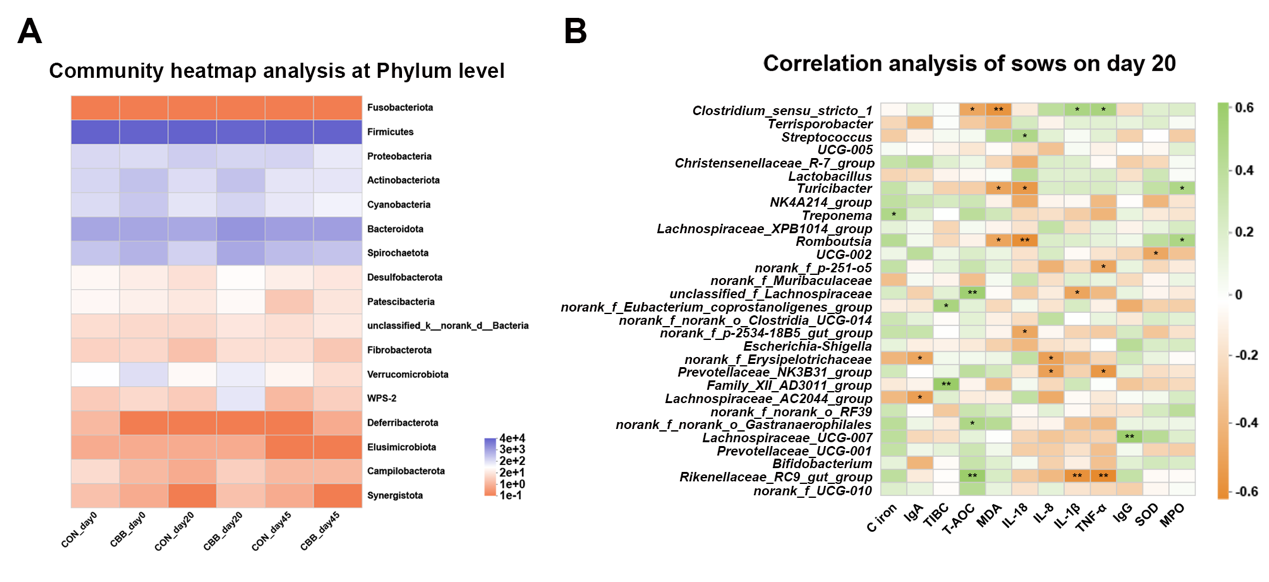


**Fig. S1**  Heatmap cluster analysis of sow fecal microbiota at the phylum level and correlations between sow fecal microbiota and serum parameters on day 20. (**A)** Heatmap cluster analysis shows the relative abundance of fecal bacteria at the phylum level in 2 groups of sows at 3 time points. (**B)** Correlations between fecal microbiota and serum parameters on day 20 in sows based on Pearson coefficient. CON_day0, the feces of sows fed a basal diet on day 0; CBB_day0, the feces of sows fed a basal diet plus CBB-mix on day 0; CON_day20, the feces of sows fed a basal diet on day 20; CBB_day20, the feces of sows fed a basal diet plus CBB-mix on day 20; CON_day45, the feces of sows fed a basal diet on day 45; CBB_day45, the feces of sows fed a basal diet plus CBB-mix on day 45. C iron=serum iron concentration; IgA=immunoglobulin A; TIBC=total iron-binding capacity; T-AOC=total antioxidant capacity; MDA=malondialdehyde; IL-18=interleukin 18; IL-8=interleukin 8; IL-1β=interleukin 1β; TNF-α=tumor necrosis factor α; IgG=immunoglobulin G; SOD=superoxide dismutase; MPO=myeloperoxidase. *n*=6. *, *P*<0.05; **, *P*<0.01.


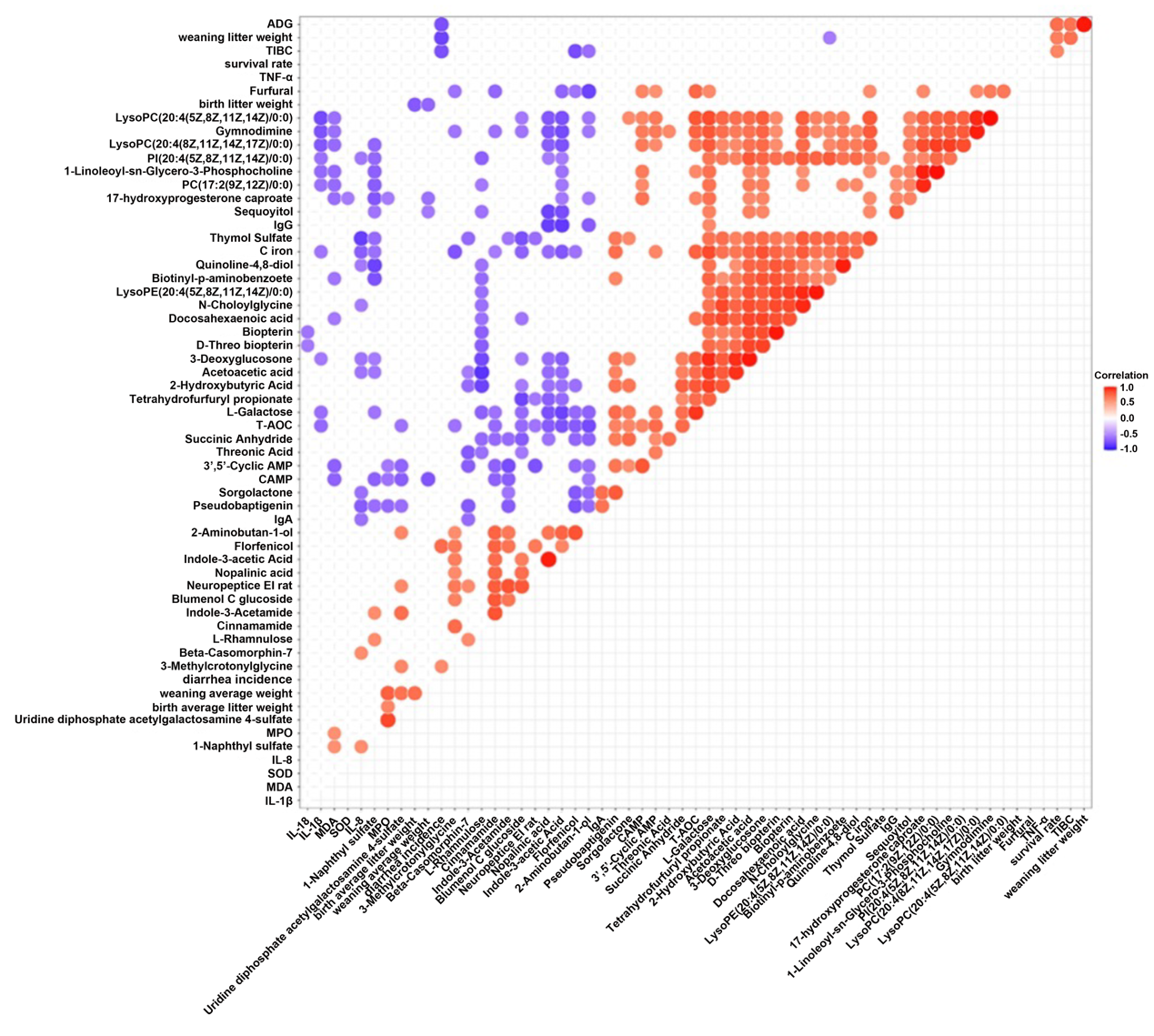


**Fig. S2**  The correlation between colostrum metabolome and phenotypic data in piglets. The serum data were from piglets at 1 day of age. Diarrhea incidence, average litter weight, litter weight at birth, survival rate, and weaning litter weight were from piglets at 28 days of age. C iron=serum iron concentration; IgA=immunoglobulin A; TIBC=total iron-binding capacity; T-AOC=total antioxidant capacity; MDA=malondialdehyde; IL-18=interleukin 18; IL-8=interleukin 8; TNF-α=tumor necrosis factor α; IL-1β=interleukin 1β; IgG=immunoglobulin G; SOD=superoxide dismutase; MPO=myeloperoxidase. Blank indicates no correlation (*P*>0.05).


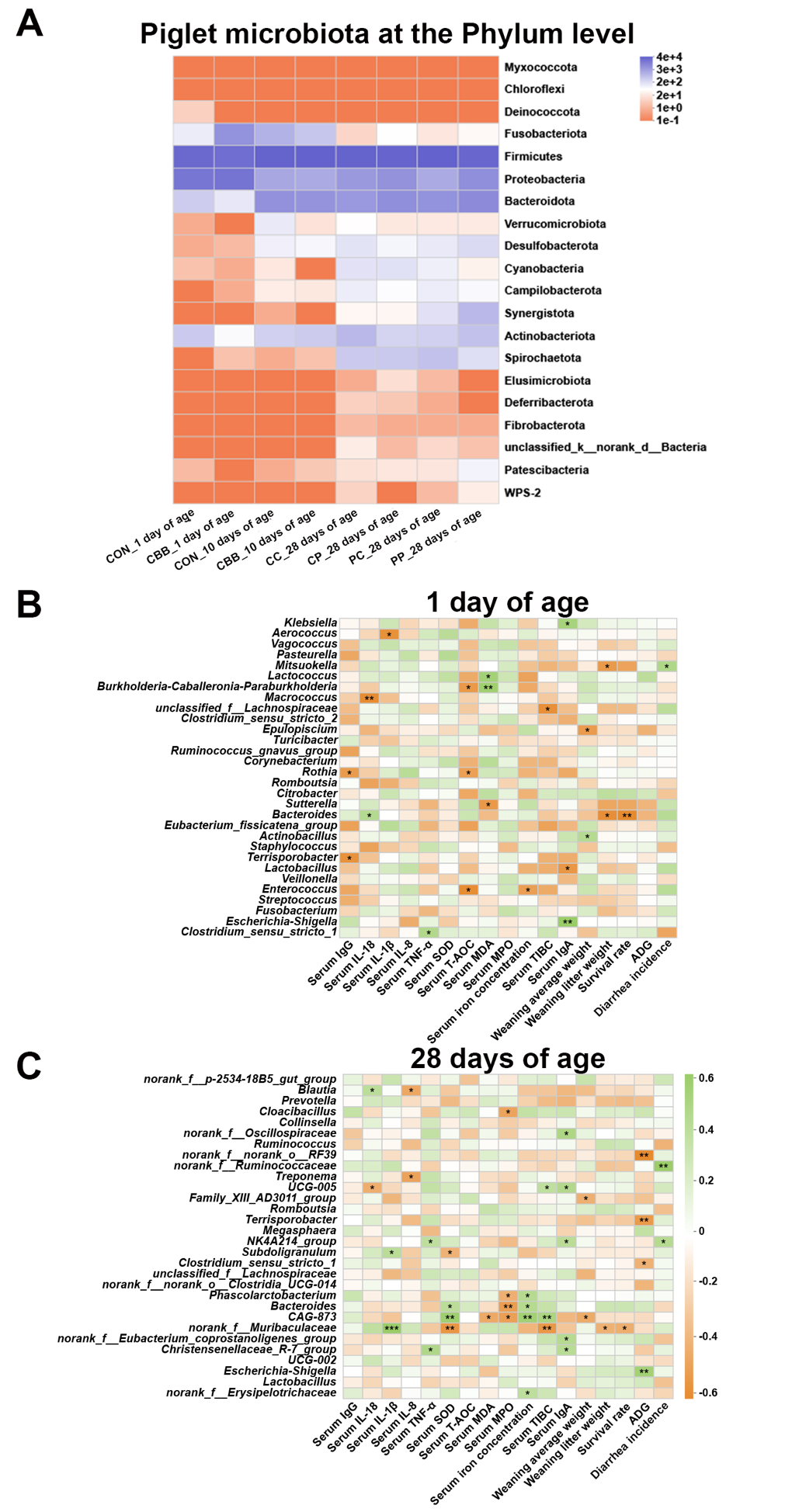


**Fig. S3** Heatmap cluster analysis of piglet fecal microbiota at the phylum level and correlations between piglet fecal microbiota and serum parameters at 1 and 28 days of age. **(A)** Heatmap cluster analysis shows the relative abundance of fecal bacteria at the phylum level in piglets at different time. (**B)** Correlations between fecal microbiota and serum parameters in piglets at 1 day of age based on Pearson coefficient. (**C)** Pearson coefficient-based correlations between the fecal microbiota and serum indicators in piglets at 28 days of age. CON_1 day of age, the feces of the offspring piglets with sows fed a basal diet at 1 day of age; CBB_1 day of age, the feces of the offspring piglets with sows fed a basal diet plus CBB-mix at 1 day of age; CON_10 days of age, the feces of the offspring piglets with sows fed a basal diet at 10 days of age; CBB_10 days of age, the feces of the offspring piglets with sows fed a basal diet plus CBB-mix at 10 days of age; CC_28 days of age, the feces of the offspring piglets with sows and piglets fed a basal diet at 28 days of age; CP_28 days of age, the feces of the offspring piglets with sows fed a basal diet plus CBB-mix and piglets fed basal diet at 28 days of age; PC_28 days of age, the feces of the offspring piglets with sows fed a basal diet and piglets fed basal diet plus CBB-mix at 28 days of age; PP_28 days of age, the feces of the offspring piglets with sows and piglets fed a basal diet plus CBB-mix at 28 days of age. C iron=serum iron concentration; IgA=immunoglobulin A; TIBC=total iron-binding capacity; T-AOC=total antioxidant capacity; MDA=malondialdehyde; IL-18=interleukin 18; IL-8=interleukin 8; TNF-α=tumor necrosis factor α; IL-1β=interleukin 1β; IgG=immunoglobulin G; SOD=superoxide dismutase; MPO=myeloperoxidase. *n*=6. *, *P*<0.05; **, *P*<0.01; ***, *P*<0.001.
